# Deubiquitylase OTUD6B Governs pVHL Stability in an Enzyme‐Independent Manner and Suppresses Hepatocellular Carcinoma Metastasis

**DOI:** 10.1002/advs.201902040

**Published:** 2020-03-08

**Authors:** Xinxin Liu, Xiaoli Zhang, Zhiqiang Peng, Chunnan Li, Ze Wang, Chanjuan Wang, Zhikang Deng, Bo Wu, Yu Cui, Zhanxin Wang, Chun‐Ping Cui, Min Zheng, Lingqiang Zhang

**Affiliations:** ^1^ State Key Laboratory of Proteomics Beijing Proteome Research Center National Center for Protein Sciences (Beijing) Beijing Institute of Lifeomics Beijing 100850 China; ^2^ Key Laboratory of Cell Proliferation and Regulation Biology of Ministry of Education College of Life Sciences Beijing Normal University Beijing 100875 China; ^3^ State Key Laboratory for Diagnosis and Treatment of Infectious Diseases The First Affiliated Hospital School of Medicine Zhejiang University Hangzhou 310000 China; ^4^ Collaborative Innovation Center for Diagnosis and Treatment of Infectious Diseases Hangzhou 310000 China

**Keywords:** deubiquitylase, hepatocellular carcinoma metastasis, hypoxia inducible factor, OTUD6B, von Hippel–Lindau

## Abstract

Hypoxia inducible factors (HIFs) are the key transcription factors that allow cancer cells to survive hypoxia. HIF's stability is mainly controlled by von Hippel–Lindau (pVHL)‐mediated ubiquitylation. Unlike sporadic clear‐cell renal carcinomas, *VHL* mutation is rarely observed in hepatocellular carcinoma (HCC) and the regulatory mechanisms of pVHL‐HIF signaling remain elusive. Here, it is shown that deubiquitylase ovarian tumor domain‐containing 6B (OTUD6B) suppresses HCC metastasis through inhibiting the HIF activity. OTUD6B directly interacts with pVHL, decreases its ubiquitylation and proteasomal degradation to reduce HIF‐1α accumulation in HCC cells under hypoxia. Surprisingly, OTUD6B limits the ubiquitylation of pVHL independent of its deubiquitylase activity. OTUD6B couples pVHL and elongin B/C to form more CBC^VHL^ ligase complex, which protects pVHL from proteasomal degradation. Depletion of OTUD6B results in the dissociation of CBC^VHL^ complex and the degradation of pVHL by Trp Asp repeat and suppressors of cytokine signaling box‐containing protein 1 (WSB1). In human HCC tissues, the protein level of OTUD6B is positively correlated with pVHL, but negatively with HIF‐1α and vascular endothelial growth factor. Low expression of OTUD6B predicts poor patient survival. Furthermore, *OTUD6B* gene is a direct transcriptional target of HIF‐1α and upregulated upon hypoxia. These results indicate a previously unrecognized feedback loop consisting of OTUD6B, pVHL, and HIF‐1α, and provide insights into the targeted hypoxic microenvironment for HCC therapy.

## Graphical abstract

In this study, it is revealed that deubiquitylase ovarian tumor domain‐containing 6B (OTUD6B) couples von Hippel–Lindau (VHL) and elongin B/C to form more Cul2‐elongin B/C‐VHL ligase complex, which protects VHL protein from proteasomal degradation and represses the hypoxia inducible factor (HIF) pathway. Furthermore, OTUD6B is identified as a direct target of HIF‐1α and the negative feedback loop between OTUD6B and HIF‐1α regulates liver cancer metastasis under hypoxia.

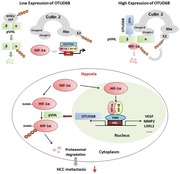

## 1 Introduction

Liver cancer is the sixth most commonly diagnosed cancer and the fourth leading cause of cancer death, with about 841 000 new cases and 782 000 deaths annually worldwide.^[^ ^1^ ^]^ Hepatocellular carcinoma (HCC) is the major form of primary liver cancer and particularly prevalent in Southeast Asia including regions of China and the 5‐year survival of HCC patients is less than 10%.^[^ ^2^ ^]^ Most HCC patients present symptoms at advanced stages when metastasis has already occurred. Metastasis not only limits the treatment option for HCC, it is the main cause of liver and organ failure, as well as tumor recurrence.^[^ ^2^ ^]^

Hypoxia is a common feature of a solid tumor's microenvironment and regulates approximately 1% of the genes that play a role in the signaling pathways that control various aspects of tumor progression.^[^ ^3^ ^]^ Hypoxia inducible factor‐1 (HIF‐1) is a master regulator of oxygen homeostasis with pleiotropic effects. It is a heterodimeric transcription factor composed of two subunits—an oxygen‐regulated subunit HIF‐1α and a stable constitutively expressed nuclear factor, HIF‐1β.^[^ ^4^ ^]^ Besides HIF‐1α, there are two other transcription factors in this family of proteins, HIF‐2α and HIF‐3α. The regulation and function of HIF‐3α is not yet entirely clear, and it is the least‐studied protein of these factors. HIF‐2α, also known as Endothelial PAS domain‐containing protein 1 (EPAS1), is highly homologous to HIF‐1α.^[^ ^5^ ^]^ Despite the similarities, HIF‐2α has several different transcriptional targets from HIF‐1α, which can also be activated during severe hypoxia.^[^ ^6^ ^]^ In HCC, HIF‐1α and HIF‐2α are closely associated with poor prognosis of patients, and HIF‐1α expression is high in human HCCs that have venous metastasis and lymph node metastasis.^[^ ^7^ ^]^ Recently, HIF‐1α and HIF‐2α were revealed to be able to enhance cancer stemness, increase liver cancer stem cell subpopulations and promote hepatocarcinogenesis and metastasis.^[^ ^8^ ^]^

Under normoxic conditions, prolyl hydroxylases (PHDs) catalyze the hydroxylation of proline residues within oxygen‐dependent degradation domains (ODD) of HIF‐α, which are recognized by the von Hippel–Lindau (VHL) E3 ubiquitin ligase complex, leading to HIF‐α ubiquitination and subsequent degradation.^[^ ^9^, ^10^ ^]^ Owing to its crucial role in HIF‐α degradation, loss‐of‐function mutations in the *VHL* gene result in constitutive activation of HIF signaling and are characteristic of several cancer syndromes, including clear cell renal cell carcinoma.^[^ ^10^ ^]^ In addition to regulation by prolyl hydroxylation, oxygen‐dependent hydroxylation of a key asparagine residue by factor inhibiting HIF (FIH) disrupts the binding of the p300 transcriptional coactivator to HIF, thereby inhibiting its transcriptional activation potential.^[^ ^11^ ^]^ Oxygen‐dependent hydroxylases provide an elegant oxygen sensing mechanism that directs the transcriptional response to hypoxia.

Protein VHL (pVHL)‐dependent ubiquitin‐proteasomal degradation is the main regulatory mechanism for the stability of HIF‐1α protein.^[^ ^12^ ^]^ pVHL is the substrate recognition component of Cullin‐RING ubiquitin ligase complex that includes elongin B, elongin C, Rbx1, and Cullin 2, known as Cul2‐elongin B/C (CBC) complex.^[^ ^13^, ^14^ ^]^ *VHL* germline mutation often results in *VHL* syndrome and is characterized by development of various tumors, including renal clear cell carcinomas and other highly vascularized tumors.^[^ ^15^ ^]^ In sporadic clear‐cell renal carcinomas (ccRCCs), the somatic mutation and inactivation frequency of *VHL* is up to 50%.^[^ ^16^, ^17^ ^]^ The loss of pVHL leads to HIF accumulation and translocation into the nucleus, which subsequently activates the transcription of HIF target genes related to critical oncogenic pathways. In HCC, however, although pVHL have also been demonstrated to function as tumor suppressor,^[^ ^18^, ^19^, ^20^ ^]^ very rare somatic mutation in *VHL* is observed.^[^ ^21^, ^22^ ^]^ The mechanisms underlying the regulation of wild type pVHL in HCC cells remain elusive.

Previous studies showed that pVHL has a rapid protein turnover rate ^[^ ^23^ ^]^ and ubiquitin‐proteasome system (UPS) mediated degradation plays a critical role in controlling pVHL stability.^[^ ^24^, ^25^ ^]^ Ubiquitylation is a dynamic and reversible process coordinated by the action of ubiquitylating and deubiquitylating enzymes. The conjugation of ubiquitin to proteins is catalyzed by ubiquitin‐activating enzyme (E1), ubiquitin‐conjugating enzyme (E2), and ubiquitin ligase (E3).^[^ ^26^ ^]^ Conversely, ubiquitin removal is catalyzed by deubiquitylases (DUBs), which specifically cleave the isopeptide or peptidic bond and remove ubiquitin from the targeted proteins.^[^ ^27^ ^]^ Several ubiquitylating enzymes have been reported to be involved in pVHL stability regulation. E2‐EPF ubiquitin carrier protein (UCP), one of member of E2 enzyme family, forms a complex with pVHL and catalyzes an E3‐independent ubiquitylation and subsequent destruction of pVHL.^[^ ^24^, ^28^, ^29^, ^30^ ^]^ Additionally, Trp Asp repeat and suppressors of cytokine signaling box‐containing protein 1 (WSB1), a newly identified E3 ligase for pVHL, has been demonstrated to promote cancer invasion and metastasis through targeting pVHL.^[^ ^25^ ^]^ Nevertheless, the DUB responsible for removing the ubiquitin linkage of pVHL has not been identified.

In this study, we show that ovarian‐tumor (OTU) domain‐containing protein 6B (OTUD6B), a member of OTU deubiquitylating enzyme family, inhibits the activation of HIF pathway via maintaining the protein stability of pVHL and thus functions as a tumor suppressor for HCC metastasis. Further we reveal that OTUD6B interacts with pVHL and reduces the ubiquitylation of pVHL in an enzyme‐independent manner. OTUD6B couples pVHL and elongin B/C subunits to form more CBC^VHL^ ligase complex which protects pVHL from degradation. Interestingly, we also found that *OTUD6B* gene is a direct transcriptional target of HIF‐1α in HCC cells. These findings suggest a negative feedback loop among OTUD6B, pVHL, and HIF‐1α, which regulates HCC metastasis under hypoxia.

## 2 Results

### 2.1 OTUD6B Suppresses HCC Metastasis

DUBs have been documented to play fundamental roles in human cancer through their ability to specifically deconjugate ubiquitin from targeted proteins. To delineate the roles of DUBs in HCC development, we first assessed the mRNA levels of the 98 members of DUB family in human HCC tissues and their corresponding non tumorous liver tissues (NT‐Ls) in the cancer genome atlas data of National Cancer Institute, USA. We observed a significant upregulation of most examined OTU subfamily DUBs, including OTUB2, OTUD7A, OTUD6B, OTUD3, OTUD7B, ALG13, OTUB1, and OTULIN (Table S1 and Figure S1A, Supporting Information). Additionally, the results of expression analysis on the data of Chinese Human Proteome Project (CNHPP) ^[^ ^31^ ^]^ (110 paired tumor and nontumor tissues of clinical early‐stage HCC) showed consistently upregulation of protein level of OTUs (including OTUD7B, OTUD6B, ALG13, OTUB1, OTULIN) in HCC tissues (Table S2 and Figure S1B, Supporting Information).

**Figure 1:**
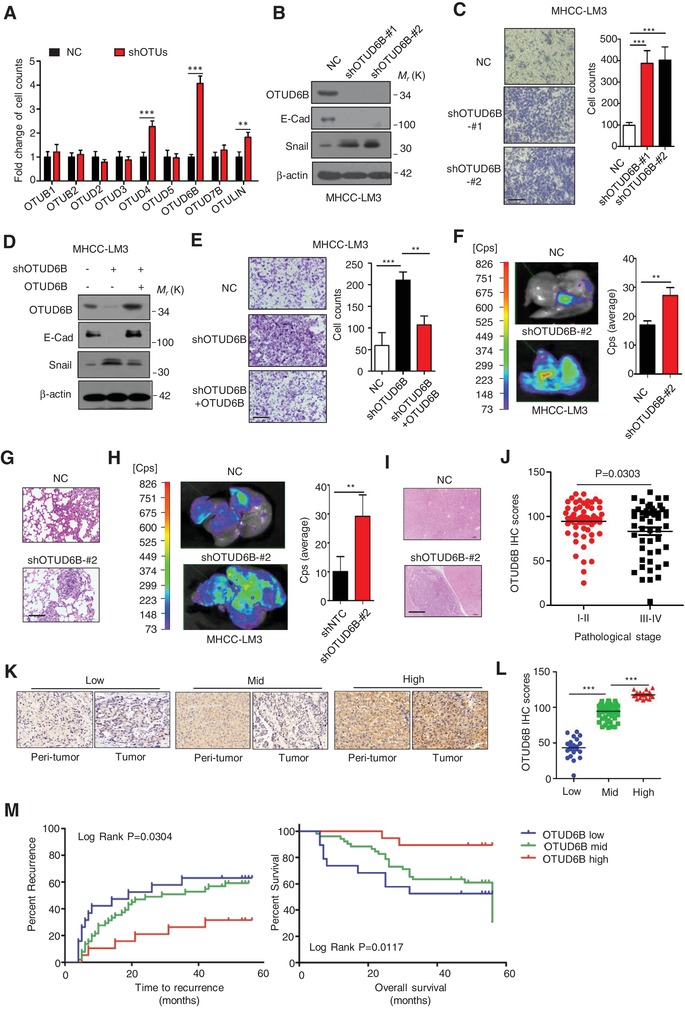
OTUD6B suppresses HCC metastasis and its low level predicts poor survival in HCC patients. A) The lentivirus with shRNA targeting indicated OTU DUBs were each infected into MHCC‐LM3 cells and generated HCC cell lines with stable knockdown of indicated OTUs. Transwell assays were conducted. Results are shown as mean ± s.d. *n* = 3 independent experiments. ^**^ *P* < 0.01, ^***^ *P* < 0.001, Student's *t* test. B,D) Immunoblot assays were conducted with indicated antibodies in HCC cells (MHCC‐LM3) with OTUD6B stable knockdown. C,E) Transwell assay was conducted in HCC cells (MHCC‐LM3) with OTUD6B stable knockdown (scale bar, 50 µm). Results are shown as mean ± s.d. *n* = 3 independent experiments. ^**^ *P* < 0.01, ^***^ *P* < 0.001, Student's *t* test. F,H) 1×10^6^ Luciferase‐expressing HCC cells were injected into nude mice by tail vein. The mice were euthanized 8 weeks later by a cervical dislocation. F) Lung and H) liver tissues were isolated for analysis of IVIS imaging. Results are shown as mean ± s.d. *n* = 5 independent experiments. ^**^ *P* < 0.01, Student's *t* test. Paraffin‐embedded sections of G) lung and I) liver tissues of nude mice were performed. Histology hematoxylin and eosin (H&E) staining was used to check formation of metastasis (scale bar, 100 µm). J) Immunohistochemistry (IHC) was performed with OTUD6B antibody on human HCC tissues. H‐score was calculated using the methods described in the Experimental Section. The scatter plot was used to show the OTUD6B IHC score of human HCC samples with pathological stage I–II or III–IV. K) Representative images from IHC staining of OTUD6B in HCC (scale bar, 100 µm). L) The scatter plot of OTUD6B IHC score in HCC samples with low, median and high OTUD6B level. ^***^ *P* < 0.001, Student's *t* test. M) The Kaplan–Meier curves of OTUD6B in HCC for both overall survival (OS) and time to recurrence.

To determine the effects of OTUs in HCC metastasis, we generated MHCC‐LM3 HCC cells with stable knockdown of each OTU member including OTUB1, OTUB2, OTUD2, OTUD3, OTUD4, OTUD5, OTUD6B, OTUD7B, and OTULIN. We observed that OTUD6B knockdown increased cell migration capacity by more than four folds (**Figure** **1**A–C; Figure S1C–O, Supporting Information), but had no significant effect on cell proliferation (Figure S1P, Supporting Information). Consistently, in HCC cells with OTUD6B depletion, the protein level of E‐cadherin, one of epithelial marker,^[^ ^32^ ^]^ was dramatically reduced, while the level of Snail, the mesenchymal marker,^[^ ^33^ ^]^ was increased markedly (Figure 1B; Figure S1M, Supporting Information). Subsequently, we reintroduced OTUD6B into HCC cells with endogenous OTUD6B depletion, the levels of E‐cadherin and Snail and the capacity of cell migration were restored (Figure 1D,E). Further, a nude mouse tail vein metastasis model was used to assess the metastatic ability of MHCC‐LM3 cells in vivo. We found that OTUD6B depletion significantly enhanced the tumor metastases of lung (Figure 1F,G) and liver (Figure 1H,I).

Additionally, we determined the possible effect of OTUD6B on angiogenesis in tumors. We generated subcutaneous xenograft nude mouse model and estimated the angiogenesis in tumors. As shown in Figure S1Q,R (Supporting Information), we observed that OTUD6B knockdown had no influence on tumor growth, but the number of blood vessels indicated by CD31 expression was significantly increased in the xenograft tumors with OTUD6B knockdown, compared with the negative control.

OTUD6B was widely expressed in various HCC cell lines (Figures S1S,T, Supporting Information). To determine the clinicopathological correlation of OTUD6B, we conducted immunohistochemical (IHC) staining on 90 pairs of human HCCs and their corresponding NT‐Ls. We found that OTUD6B level had a significant decreasing trend with the increase of HCC pathological stage (including I–II, III–IV stage, *P* = 0.0303) (Figure 1J; Table S3, Supporting Information). Further, we divided all the HCC tissues into 3 groups (low, middle, and high) according to OTUD6B level as shown in Figure 1K,L. Kaplan–Meier curves were measured to analyze the correlation of OTUD6B level with survival and recurrence time. The results showed that low level of OTUD6B predicted a shorter survival time for overall survival (OS) and disease‐free survival (DFS) (Figure 1M). Together, these results suggest that OTUD6B is a suppressor factor of HCC metastasis.

### 2.2 OTUD6B Negatively Regulates HIF‐1α Pathway and Increases HIF‐1α Ubiquitylation

To explore the mechanism underlining OTUD6B suppressing HCC metastasis, we performed RNA sequencing (RNA‐seq) in HCC cells (MHCC‐LM3) with OTUD6B depletion and negative nontargeting control (NTC). Total 110 differentially expressed genes (DEGs) were identified by RNA‐seq including 90 upregulated and 20 downregulated genes in OTUD6B depletion cells (Figure S2A and Table S4, Supporting information). Further analysis with gene ontology (GO) and KEGG pathway indicates that these DEGs were enriched for cell motility, cell adhesion molecules and tumor environment (Figure S2B, Supporting information). Interestingly, the RNA‐seq data suggested that depletion of OTUD6B remarkably activated HIF pathway in HCC cells (Figure S2C,D, Supporting Information).

**Figure 2:**
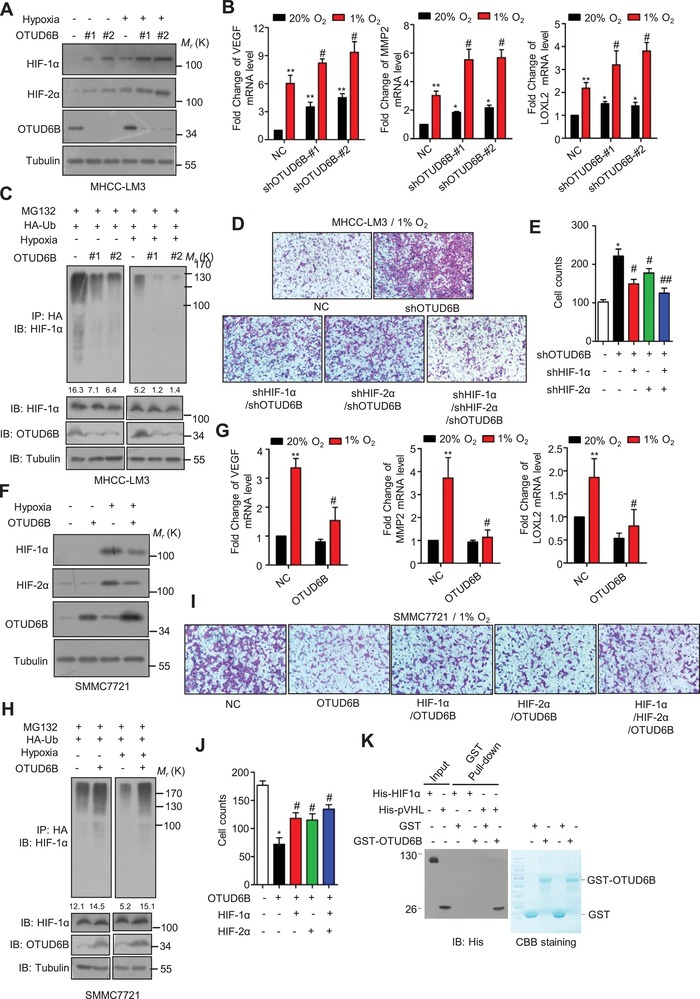
OTUD6B negatively regulates HIF pathway. A) Immunoblotting of HIF‐1α, HIF‐2α and OTUD6B in MHCC‐LM3 cells transfected with indicated shRNA. B) Q‐PCR was used to examine the mRNA level of HIF target genes in MHCC‐LM3 cells transfected with indicated shRNA. C) HA‐Ub was cotransfected together with indicated shRNA into HEK293T cells. Cells were treated with MG132 for 8 h before collection. Then HA‐Ub was immunoprecipitated with anti‐HA antibody and immunoblotted with anti‐HIF‐1α antibody. D) Transwell assay was performed in HCC cells transfected with indicated shRNA (scale bar, 50 µm). E) The means of migrated cells in (D) were shown as the column chart. F) Immunoblotting of HIF‐1α, HIF‐2α, and OTUD6B in SMMC7721 cells with stable OTUD6B overexpression. G) Q‐PCR was used to examine the mRNA level of HIF target genes in SMMC7721 cells with stable ectopic OTUD6B overexpression. H) HA‐Ub was cotransfected together with indicated constructs into HEK293T cells. Cells were treated with MG132 for 8 hours before collection. Then HA‐Ub was immunoprecipitated with anti‐HA antibody and immunoblotted with anti‐HIF‐1α antibody. I) Transwell assay was performed in HCC cells transfected with indicated constructs (scale bar, 50 µm). J) The means of migrated cells in (I) were shown as the column chart. K) The purified His‐pVHL or His‐HIF‐1α was incubated respectively with purified GST or GST‐OTUD6B. The mixtures were subjected to GST pull down and blotted. Results are shown as mean ± s.d. *n* = 3 independent experiments. ^*^ *P* < 0.05, ^**^ *P* < 0.01 compared with NC group, ^#^ *P* < 0.05, ^##^ *P* < 0.01 compared with group of OTUD6B knockdown or ectopic OTUD6B overexpression, Student's *t* test.

We further validated the influence of OTUD6B on HIF signaling using a series of experiments. We found that in OTUD6B knockdown HCC cells (MHCC‐LM3), the protein levels but not mRNA levels of HIF‐1α and HIF‐2α were markedly increased under both hypoxia and normoxia (**Figure** **2**A; Figure S2E, Supporting information). Functionally, we tested the effect of OTUD6B knockdown on cell migration under normoxia and hypoxia. Compared with negative control, increased cell migration ability was observed in MHCC‐LM3 cells with OTUD6B knockdown under both hypoxia and normoxia (Figure S2F, Supporting Information). We assessed the transcriptional factor activity of HIF‐1α on vascular endothelial growth factor (VEGF) gene by reporter gene assay using a 6×HRE (hypoxic response element) VEGF promoter driven‐luciferase reporter. OTUD6B depletion increased hypoxia‐induced activation of VEGF promoter (Figure 2G, Supporting information) and the mRNA levels of VEGF, MMP2 and LOXL2 in HCC cells (Figure 2B). Using immunoprecipitation (IP) assay, we observed that OTUD6B knockdown decreased HIF‐1α ubiquitylation (Figure 2C) in cells. Further, blockage of HIF pathway by depletion of HIF‐1α, HIF‐2α, or combined HIF‐1α and HIF‐2α abolished the effect of OTUD6B knockdown on cell migration (Figure 2D,E; Figure S2H, Supporting Information).

Consistently, ectopic expression of OTUD6B decreased the protein levels of HIF‐1α and HIF‐2α, but not the mRNA levels (Figure 2F; Figure S2I, Supporting Information) under hypoxia. Decreased cell migration ability was observed in SMMC7721 cells with ectopic OTUD6B overexpression under both hypoxia and normoxia (Figure S2J, Supporting Information). Overexpression of OTUD6B decreased the mRNA levels of VEGF, MMP2, and LOXL2 in HCC cells (Figure 2G) and hypoxia‐induced activation of VEGF promoter (Figure 2K, Supporting Information). Similarly, we observed that overexpression of ectopic OTUD6B increased the HIF‐1α ubiquitylation under hypoxia (Figure 2H). Moreover, we found that activation of HIF pathway by ectopic expression of HIF‐1α, HIF‐2α, or combined HIF‐1α and HIF‐2α rescued the inhibitory effect of OTUD6B on cell migration (Figure 2I,J; Figure S2L, Supporting Information). These results indicate that OTUD6B is a negative regulator of HIF‐1α activity.

Then we asked if OTUD6B interact with HIF‐α. To address this issue, we performed the GST pull‐down assay to detect the interaction between OTUD6B and HIF‐1α. As shown in Figure 2K, we observed that OTUD6B was unable to bind to HIF‐1α in cell‐free system, suggesting that HIF‐1α is not the direct target of OTUD6B. Interestingly, we observed that OTUD6B was able to interact with pVHL, the E3 ligase for HIF‐1α, implying that OTUD6B might suppress HIF pathway via regulating pVHL in HCC under hypoxia.

### 2.3 OTUD6B Stabilizes pVHL in an Enzyme‐independent Manner

Considering pVHL is able to interact with OTUD6B and promote the HIF‐1α ubiquitylation and subsequent degradation, we wondered if OTUD6B destabilizes HIF‐1α via removal of ubiquitin conjugation of pVHL. Previous studies have demonstrated that E2‐EPF UCP ^[^ ^24^ ^]^ and WSB1 ^[^ ^25^ ^]^ play as the E2 conjugation enzyme and E3 ligase for pVHL degradation respectively. So far, no DUB has been identified to remove the ubiquitin linkage of pVHL and stabilize pVHL, although two DUBs, VDU1 and VDU2 (pVHL‐interacting deubiquitinating enzyme‐1 and ‐2) have been demonstrated to interact with pVHL.^[^ ^34^, ^35^ ^]^

**Figure 3:**
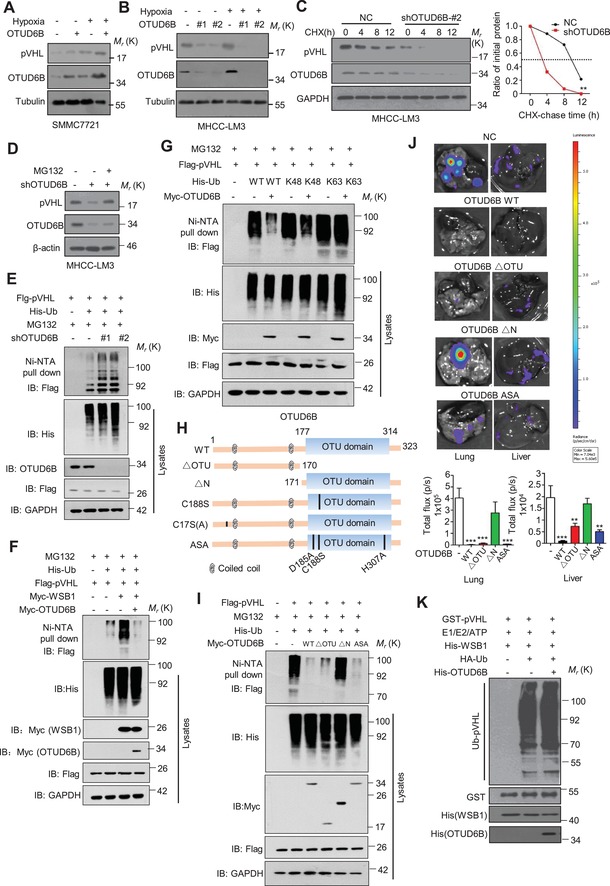
OTUD6B stabilizes pVHL in an enzyme‐independent manner. A) Immunoblotting of pVHL in SMMC7721 cells with stable OTUD6B overexpression. B) Immunoblotting of pVHL in MHCC‐LM3 cells transfected with indicated shRNA. C) HCC cells were treated with 10 µg mL^−1^ cycloheximide (CHX), and collected at the indicated times for western blot. Quantification of pVHL levels relative to GAPDH is shown. Results are shown as mean ± s.d. *n* = 3 independent experiments. ^∗∗^ *P* < 0.01, two‐way ANOVA test. D) Immunoblotting of pVHL in MHCC‐LM3 cells with shOTUD6B with or without MG132 treatment. E–G,I) His‐Ub was cotransfected together with indicated shRNA into HEK293 cells. Cells were treated with MG132 for 8 h before collection. Then His‐Ub was pulled down by Ni‐NTA and immunoblotted with anti‐flag antibody. H) Overview of the structures of OTUD6B wild type and different truncates. J) 1×10^6^ Luciferase‐expressing SMMC7721 HCC cells with stably overexpressing indicated constructs were injected into nude mice by tail vein. The mice were euthanized 8 weeks later by a cervical dislocation. Lung (left) and liver (right) tissues were isolated for analysis of IVIS imaging. Results are shown as mean ± s.d. *n* = 5 independent experiments. ^**^ *P* < 0.01, ^***^ *P* < 0.001 Student's *t* test. K) Cell‐free pVHL ubiquitylation assay. The purified GST‐pVHL proteins were incubated with His‐WSB1 as E3, commercial E1, E2, and HA‐Ub for 2 h at 37 °C. The mixtures were subjected to GST pull‐down and western blot with anti‐HA antibody.

Firstly, we examined the effect of OTUD6B on pVHL stability. The results showed that ectopic expression of OTUD6B elevated pVHL protein level but not its mRNA level (**Figure** **3**A; Figure S3A, Supporting Information), while OTUD6B silence decreased pVHL protein level without significant effect on its mRNA level (Figure 3B; Figure S3B–D, Supporting Information). The half‐life of pVHL was shortened in cells with OTUD6B depletion (Figure 3C; Figure S3E, Supporting Information). Treatment with MG132, the inhibitor of proteasome, restored the pVHL level comparable to HCC cells without shOTUD6B treatment (Figure 3D; Figure S3F, Supporting Information), indicating that the pVHL was undergoing proteasome‐dependent degradation in HCC cells with OTUD6B knockdown. Additionally, we observed that shRNA targeting OTUD6B increased pVHL ubiquitylation (Figure 3E), and ectopic expression of OTUD6B suppressed pVHL ubiquitylation induced by WSB1 in cells (Figure 3F). Further, we showed that OTUD6B effectively reduced Lys 48‐linked polyubiquitylation, but not the nondegradative Lys 63‐linked polyubiquitylation of pVHL (Figure 3G).

OTUD6B protein contains an N‐terminal region (including two coiled‐coil motifs) and a C‐terminal catalytic OTU domain. Bioinformatic analysis suggests the D185, C188, and H307 in the OTU domain form the triad catalytic active core. To identify the critical region or site of OTUD6B on pVHL regulation, we generated a series of OTUD6B truncates and point‐mutants as shown in Figure 3H and Figure S3G (Supporting Information). To our surprise, similar to the wild type (OTUD6B WT), deletion of OTU domain (OTUD6B ∆OTU) or point‐mutation of the putative catalytic active sites (OTUD6B ASA, C188S, and C17S(A)) were still able to suppress the ubiquitylation of pVHL in cells (Figure 3I; Figure S3H,I, Supporting Information). In contrast, deletion of the N‐terminal coiled‐coil‐containing region lost the ability to remove pVHL ubiquitylation in cultured cells (Figure 3I). Then transwell assay and nude mouse tail vein metastasis model were performed to determine the functional role of OTUD6B truncates and mutants in vitro and in vivo. As shown in Figure 3J and Figure S3J,K, Supporting Information, compared with the negative control, the ectopic overexpression of OTUD6B WT, OTUD6B ∆OTU, and OTUD6B ASA significantly suppressed cell migration both in vitro and in vivo, while OTUD6B∆N had no effect on cell migration, which is consistent with the effects of OTUD6B and its mutants on the stability of pVHL and HIF‐1α. These results suggest that the N‐terminal part of OTUD6B is both sufficient and required for OTUD6B to suppress pVHL ubiquitylation in cells and cell migration. Further, we performed in vitro ubiquitylation assay, the results showed that in cell‐free system, OTUD6B no longer reduced the ubiquitylation of pVHL which was catalyzed by the WSB1 ligase (Figure 3K). We proposed that OTUD6B inhibits pVHL ubiquitylation in an OTU‐independent manner and additional regulatory factor(s) might be required in cells.

### 2.4 OTUD6B Interacts with pVHL

**Figure 4:**
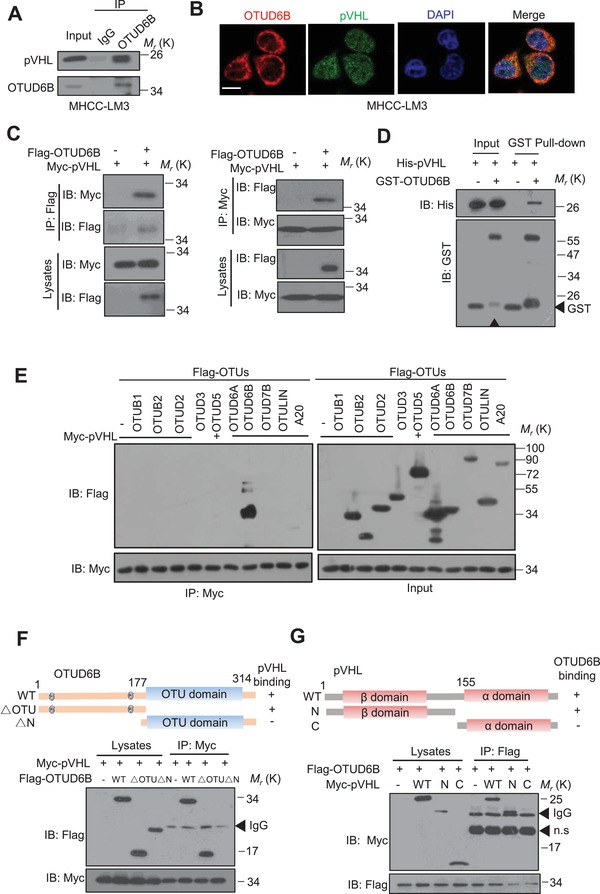
OTUD6B interacts with pVHL. A) HCC cell lysates were subject to immunoprecipitation with control IgG or anti‐OTUD6B antibodies. B) Immunofluorescence staining was performed in HCC cells with anti‐OTUD6B and anti‐pVHL antibodies to determine the colocalization of these two proteins in MHCC‐LM3 cells (scale bar, 10 µm). C) The lysates of HEK293T transfected with indicated constructs were subject to immunoprecipitation with anti‐myc (or flag) antibody. The immunoprecipitates were then blotted with anti‐flag (or myc) antibody. D) The purified his‐pVHL was incubated respectively with purified GST or GST‐OTUD6B. The mixtures were subjected to GST pull down and blotted. E) The lysates of HEK293T transfected with indicated constructs were subject to immunoprecipitation with anti‐myc antibody. The immunoprecipitates were then blotted with anti‐flag antibody. Overview of E) OTUD6B and F) pVHL structure. HEK293T cells transfected with the indicated constructs were subject to immunoprecipitation with anti‐myc antibody. The lysates and immunoprecipitates were then blotted.

Subsequently, we examined the interaction between pVHL and OTUD6B. Endogenous OTUD6B and pVHL were coimmunoprecipitated from lysates of MHCC‐LM3 cells (**Figure** **4**A). The results of immunofluorescence (IF) assay showed that pVHL and OTUD6B were colocalized in the cytoplasm of HCC cells (Figure 4B; Figure S4A, Supporting Information). Ectopic OTUD6B interacted with pVHL in HEK293 cells (Figure 4C; Figure S4B, Supporting Information). GST pull‐down assay indicated that in cell‐free system purified OTUD6B protein was able to bind to pVHL (Figure 4D). Among ten examined OTU‐type DUBs, only OTUD6B was able to interact with pVHL (Figure 4E), indicating the specificity. Deletion analysis demonstrated that the N‐terminal part, but not the C‐terminal OTU domain region of OTUD6B mediated the physical interaction with pVHL (Figure 4F), confirming the importance of the N‐terminal part of OTUD6B in pVHL regulation. Additionally, we mapped the OTUD6B‐binding region of pVHL to the N‐terminal β‐domain (Figure 4G).

### 2.5 OTUD6B Suppresses pVHL Degradation via Increasing pVHL Binding to Elongin B/C

**Figure 5:**
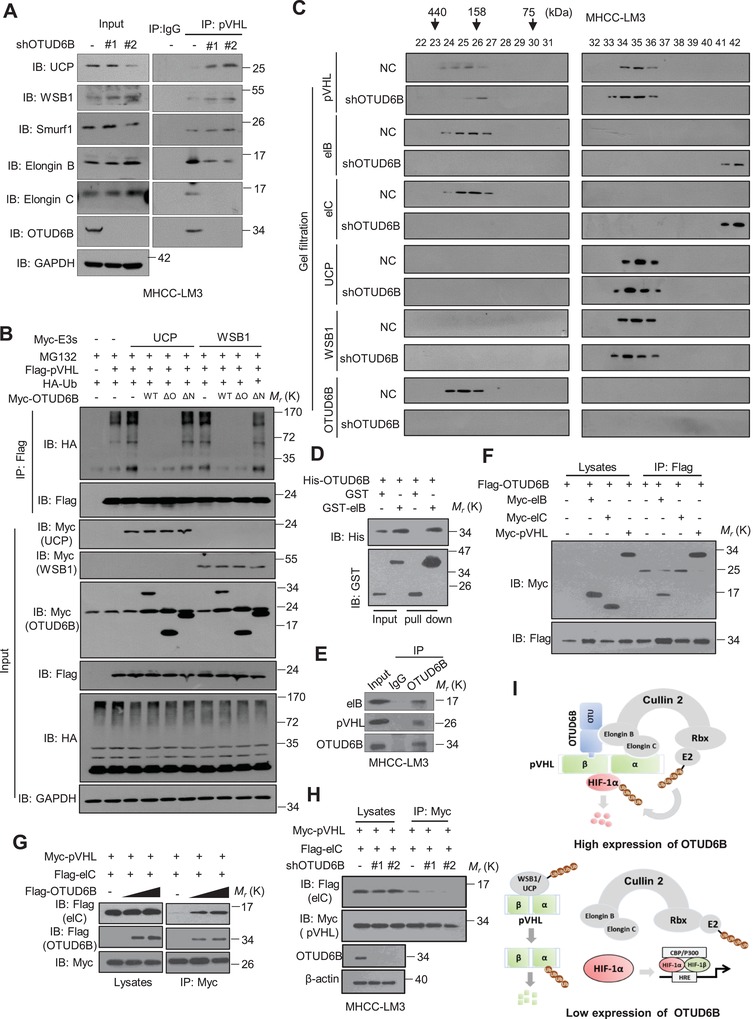
OTUD6B interacts with elongin B and enhances the binding of pVHL and elongin C. A) MHCC‐LM3 cells with NC or OTUD6B knockdown were treated with MG132 for 8 h. The lysates were subject to immunoprecipitation with control IgG or anti‐pVHL antibodies. The immunoprecipitates were then blotted with indicated antibodies. B) HA‐Ub was cotransfected together with indicated constructs in HEK293 cells. Then the lysates were subject to immunoprecipitation with anti‐flag antibody. The immunoprecipitates were then blotted with anti‐HA antibody. C) MHCC‐LM3 Cells with NTC or OTUD6B knockdown were treated with MG132 for 8 h, and then lysed in 0.5 mL of CHAPS lysis buffer. 500 µL of the lysate was loaded onto a Superdex 200 10/300 GL column. Chromatography was performed on the AKTA‐FPLC with CHAPS buffer. One column volume of eluates was fractionated with 500 mL in each fraction, at the elution speed of 0.5 mL min^−1^. 30 μL aliquots of each fraction were loaded onto SDS–PAGE gels and detected with indicated antibodies. D) The purified his‐OTUD6B was incubated respectively with purified GST or GST‐elongin B. The mixtures were subjected to GST pull down and blotted. E) HCC cell lysates were subject to immunoprecipitation with control IgG or anti‐OTUD6B antibodies. The lysates and immunoprecipitates were then blotted. F) The lysates of HEK293T cells transfected with indicated constructs were subjected to immunoprecipitation with anti‐flag. The immunoprecipitates or the eluates were then blotted. G) Increasing amounts of myc or flag‐OTUD6B were cotransfected together with indicated constructs into HEK293T cells and the lysates were subject to immunoprecipitation with anti‐myc antibody. The lysates and immunoprecipitates were then blotted. H) The lysates of HEK293T transfected with indicated shRNA and constructs were subject to immunoprecipitation with anti‐myc antibody. The lysates and immunoprecipitates were then blotted. I) The predicted work model of OTUD6B binding to CBC^VHL^ complex.

Previous studies have reported that the deubiquitylase activity of OTUD4 ^[^ ^36^, ^37^ ^]^ and OTUB1 ^[^ ^38^ ^]^ are dispensable for regulating the stability of their substrate. OTUD4 serves as a scaffold for two additional deubiquitylases USP7 and USP9X, which directly deubiquitylate human AlkB proteins.^[^ ^37^ ^]^ OTUB1 suppresses RNF168‐dependent polyubiquitylation via binding to and inhibiting UBC13 (also known as UBE2N), the cognate E2 enzyme for RNF168.^[^ ^38^ ^]^ Therefore, we speculated that OTUD6B might suppress the ubiquitylation of pVHL via influencing the interaction between pVHL and the E3 or E2 enzymes. Using Co‐IP assays, we determined the possible effect of OTUD6B on the interaction of pVHL with Smurf1,^[^ ^39^ ^]^ WSB1,^[^ ^25^ ^]^ and E2‐EPF UCP.^[^ ^24^ ^]^ As shown in Figures 5A, depletion of OTUD6B enhanced the interaction between pVHL with E2‐EPF UCP or WSB1 in HCC cells, although ectopic OTUD6B overexpression was unable to influence these interactions in cells (Figure S5A–C, Supporting Information). UbcH5c and OTUB1 were predicted as potential interacting proteins of OTUD6B.^[^ ^40^ ^]^ However, neither of them could be coimmunoprecipitated with OTUD6B from cell lysate (Figure S4D,E, Supporting Information). Additionally, we performed ubiquitylation assay in HEK293 cells and observed that ectopic OTUD6B WT and OTUD6B ∆OTU, but not OTUD6B∆N, dramatically reduced pVHL ubiquitylation induced by WSB1 or E2‐EPF‐UCP (**Figure** **5**B), suggesting that OTUD6B suppresses pVHL ubiquitylation by WSB1 or E2‐EPF‐UCP and subsequent degradation.

Then we asked why OTUD6B influences pVHL binding to E2‐EPF‐UCP or WSB1. Biochemical and structural studies revealed that pVHL forms a ternary complex with the elongin B and elongin C.^[^ ^14^, ^41^, ^42^ ^]^ Elongin C folds into an α/β roll and binds to pVHL through helices and loops at its C‐terminus.^[^ ^14^ ^]^ Elongin B/C complex prevents ubiquitylation and degradation of pVHL ^[^ ^41^, ^42^ ^]^ by E3 ligase. Wild‐type pVHL is directly stabilized by association with elongin B/C and entire pVHL‐elongin B/C complex is resistant to proteasomal degradation.^[^ ^42^ ^]^ This prompted us to examine the possible effect of OTUD6B on the interaction between pVHL and Cul2‐elongin B/C (CBC) complex. We used gel filtration chromatography assay to analyze the CBC^VHL^ complex binding of OTUD6B. As shown in Figure 5C and Figure S5F (Supporting Information), OTUD6B was detected in the same fractions with pVHL, elongin B, and elongin C, indicating that OTUD6B might be a component of CBC^VHL^ complex. Interestingly, we observed that compared with negative control, less VHL proteins were detected binding to elongin B/C in OTUD6B knockdown HCC cells treated with MG132. Additionally, we also found that OTUD6B directly bound to elongin B, but not to elongin C, in cells by both N‐terminal and C‐terminal parts (Figure 5D–F; Figure S5G–J, Supporting Information). With the increase of OTUD6B expression, the interaction between pVHL and elongin C was dramatically enhanced (Figure 5G). Consistently, the depletion of endogenous OTUD6B weakened the interaction between pVHL and elongin C (Figure 5H), indicating that OTUD6B plays a role in maintaining the stability of CBC^VHL^ complex. Above all, OTUD6B might protect pVHL from proteasomal degradation via binding to elongin B and enhancing the pVHL‐elongin C interaction (Figure 5I).

### 2.6 pVHL Mediates the Inhibitory role of OTUD6B on HCC Cell Migration

**Figure 6:**
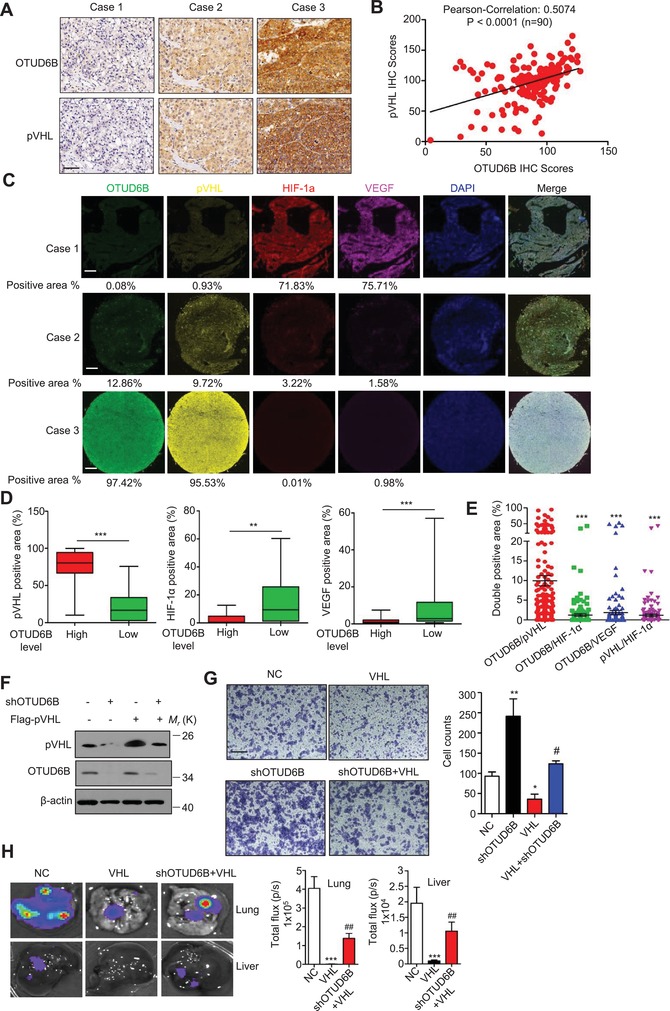
pVHL mediates the inhibitory effect of OTUD6B on HCC cell migration. A) Representative images from IHC staining of OTUD6B and pVHL in HCC tissues (*n* = 90) (scale bar, 50 µm). B) The Pearson correlation analysis between OTUD6B level and pVHL level in HCC tissues. C) Representative images from Immunofluorescence staining of OTUD6B, pVHL, HIF‐1α, and VEGF in HCC tissues (*n* = 90) (scale bar, 300 µm). D) The percentages of pVHL, HIF‐1α, or VEGF positive area in HCC tissues with high or low OTUD6B level were analyzed using the Mann–Whitney *U* test. ^*^ *P* < 0.05, ^**^ *P* < 0.01, ^***^ *P* < 0.001. E) The percentages of double positive area of indicated proteins in HCC tissues were analyzed. ^*^ *P* < 0.05, ^**^ *P* < 0.01, ^***^ *P* < 0.001, Student's *t* test. F) Immunoblotting of pVHL and OTUD6B in HCC cells with stable overexpression or knockdown of indicated genes. G) Transwell assays were performed to identify the capacity of migration in HCC cell with stable overexpression or knockdown of indicated genes. Results are shown as mean ± s.d. *n* = 3 independent experiments. ^*^ *P* < 0.05, ^**^ *P* < 0.01, ^***^ *P* < 0.001, Student's *t* test. H) 1×10^6^ Luciferase‐expressing MHCC‐LM3 cells were injected into nude mice by tail vein. The mice were euthanized 8 weeks later by a cervical dislocation. Lung and liver tissues were isolated for analysis of IVIS imaging. Results are shown as mean ± s.d. *n* = 5 independent experiments. ^*^ *P* < 0.05, ^**^ *P* < 0.01, ^***^ *P* < 0.001, Student's *t* test.

We analyzed the correlation between the protein level of OTUD6B and pVHL using our IHC results of 90 pairs of human HCCs. We observed a significant positive correlation between OTUD6B and pVHL (*P* < 0.0001, *r* ^2^ = 0.5074) (**Figure** **6**A,B). The western blots of OTUD6B and pVHL on human HCC tissues showed similar results (Figure 6A, Supporting Information). To examine the correlation of OTUD6B and HIF‐1α or HIF‐2α in HCC tissues, a tumor microarray‐based immunofluorescence was performed with antibodies against OTUD6B, pVHL, HIF‐1α, and VEGF. The results revealed that in HCC tissues, high OTUD6B expression was positively correlated with pVHL, but negatively correlated with HIF‐1α or VEGF (Figure 6C,D). The analysis of double positive area revealed that the percentage of OTUD6B^+^/pVHL^+^ cells (9.94%) in HCC tissues was much higher than that of OTUD6B^+^/HIF‐1α^+^ cells (1.37%), OTUD6B^+^/VEGF^+^ (1.73%), and pVHL^+^/ HIF‐1α^+^ (1.13%) (Figure 6E).

To further determine if the functional role of OTUD6B is dependent on the action of pVHL, we generated MHCC‐LM3 cells with stably overexpressed pVHL. Western blot confirmed the expression of pVHL in OTUD6B knockdown or NC cells (Figure 6F). Ectopic pVHL was able to repress the enhancement of cell migratory ability induced by OTUD6B knockdown in HCC cells (Figure 6G). Further, we used nude mouse tail vein metastasis model to access the metastatic ability of the HCC cells in vivo. As shown in Figure 6H, the results showed that compared with the negative control, the pVHL overexpression alone significantly suppressed cell migration, while OTUD6B knockdown partially rescues the effect of ectopic pVHL on cell metastasis. Additionally, we knocked down the expression of pVHL using lentivirus‐mediated shRNA in SMMC7721 cells which overexpressed OTUD6B (Figure 6B, Supporting Information). The depletion of pVHL repressed the decrease of cell migratory ability induced by OTUD6B overexpression (Figure 6C, Supporting Information).

We also validated this effect in human ccRCC cells (ACHN and 786‐O) and lung cancer cells (H1299). 786‐O is a pVHL‐deficient ccRCC cell line that constitutively expresses HIF‐2α.^[^ ^43^ ^]^ We knocked down OTUD6B expression in these cancer cells, and examined HIF‐2α level and the activation of Akt pathways as the downstream signals of pVHL.^[^ ^44^ ^]^ The results showed that OTUD6B depletion increased the protein level of HIF‐2α and the phosphorylation level of Akt in ACHN, H1299, and Bel7402, but not in 786‐O cells (Figure 6D, Supporting Information). Functionally, we also observed consistent results that OTUD6B depletion enhanced cell migration in ACHN, H1299, and Bel7402, but not in 786‐O cells (Figure 6E, Supporting Information).

### 2.7 OTUD6B is a Transcriptional Target Gene of HIF‐1/2 in HCC Cells

**Figure 7:**
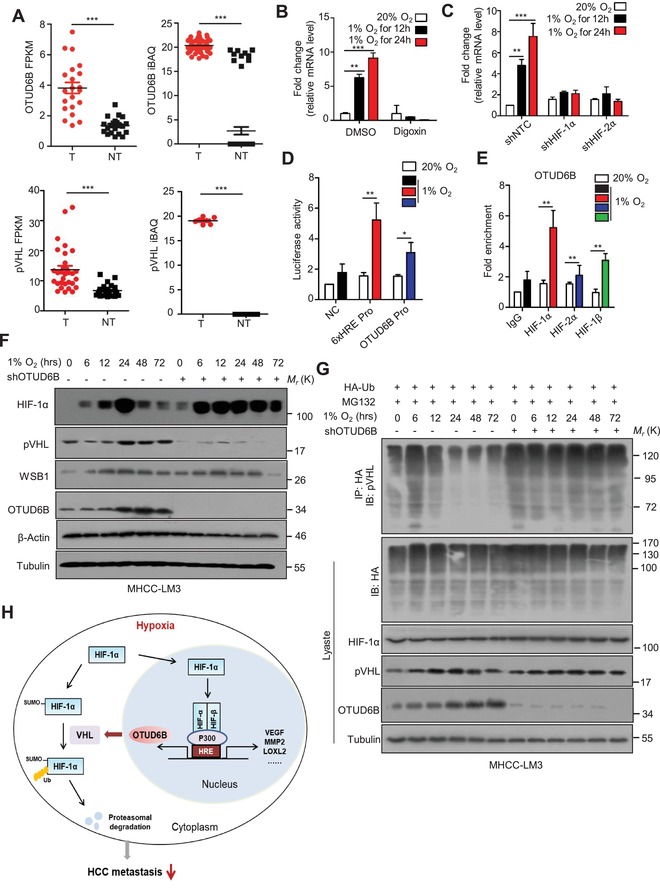
OTUD6B is a transcriptional target gene of HIF‐1α in HCC cells. A) The levels of mRNA and protein of OTUD6B and pVHL were consistently and significantly upregulated in CNHPP data on 110 pairs of human HCC tumor tissues (T) and their corresponding nontumorous livers (NT). B) Q‐PCR was used to examine the mRNA level of OTUD6B in MHCC‐LM3 cells treated with 100^−6^ m digoxin under hypoxia or normoxia. C) Q‐PCR was used to examine the mRNA level of OTUD6B in MHCC‐LM3 cells transfected with indicated shRNA under hypoxia or normoxia. D) Fold change of the relative luciferase activity was examined by luciferase‐reporter assay in SMMC‐7721 cells which were incubated under normoxia or hypoxia for 24 h. E) Chip assay was used to determine the bind of HIF‐1α, HIF‐2α and HIF‐1β to the HRE sequence in OTUD6B promoter. F) Immunoblotting of HIF‐1α, pVHL, WSB1, and OTUD6B in MHCC‐LM3 cells with stable NC or OTUD6B knockdown under hypoxia for indicated times. G) ubiquitylation of pVHL in MHCC‐LM3 cells with stable NC or OTUD6B knockdown under hypoxia for indicated times. H) The cartoon summarizing our findings. HIF‐1/2α induced by the hypoxic microenvironment increases the transcription of OTUD6B in HCC cells. OTUD6B represses the pVHL ubiquitylation and subsequent degradation, thus reducing the stability and transcriptional activity of HIF‐1α. Results are shown as mean ± s.d. *n* = 3 independent experiments. ^*^ *P* < 0.05, ^**^ *P* < 0.01, ^***^ *P* < 0.001, Student's *t* test.

The results of expression analysis on CNHPP ^[^ ^25^, ^31^ ^]^ showed that mRNA and protein levels of either OTUD6B or pVHL are significantly upregulated in human HCC tissues compared with the corresponding NT‐Ls (**Figure** **7**A; Table S2, Supporting Information). Considering the frequent hypoxic microenvironment in HCC tissues, we proposed that OTUD6B is regulated by HIF‐1/2α. Firstly, we examined the OTUD6B mRNA and protein levels 24 h post hypoxic treatment. OTUD6B expression was markedly increased in HCC cells after hypoxic treatment (Figure 7B,C; Figure S7A–C, Supporting Information). Treatment with digoxin, a well‐known HIF‐1α inhibitor,^[^ ^45^ ^]^ or HIF‐1/2α shRNA abolished these effects (Figure 7B,C; Figure S7A–C, Supporting Information). We assessed the transcriptional activity of HIF‐1α on OTUD6B using OTUD6B promoter‐driven luciferase reporter assay. Hypoxia‐induced OTUD6B transcription was observed in HCC cells (Figure 7D). Furthermore, chromatin immunoprecipitation (ChIP) assay was used to confirm the binding of HIF‐1α or HIF‐2α to OTUD6B promoter. The HRE core sequence (A/G) CGTG is found at around −786 and −24 bp in the OTUD6B promoter (Figure S7D, Supporting Information). Using the sequence of the PCR primers shown in the Supporting Information, we demonstrated that HIF‐1α, HIF‐2α, and HIF‐1β bound to the HRE sequence on the OTUD6B promoter (Figure 7E; Figure S7E, Supporting Information).

In order to explore what's happen under the relationship between OTUD6B and pVHL/HIF‐α pathway, we examined the level of HIF‐1α, OTUD6B, pVHL, and WSB1 in HCC cells treated with hypoxia for indicated times as shown in Figure 7F. HIF‐1α was induced and accumulated at 6 h post hypoxia and reached the peak at 24 h post hypoxia in HCC cells. OTUD6B was upregulated, and pVHL was decreased at 12 h post hypoxia, then pVHL increased and became stable at 24 h after hypoxia due to decreased ubiquitylation (Figure 7G). While in HCC cells with OTUD6B knockdown, HIF‐1α was dramatically increased 6 h post hypoxia and remained at high level due to the decreased protein level of OTUD6B and pVHL (Figure 7F) and increased ubiquitylation of pVHL (Figure 7G). Additionally, we examined the influence of hypoxia on the interaction between pVHL and elongin C. Under hypoxia condition, the interaction between pVHL and elongin C was enhanced and OTUD6B depletion blunted the effect (Figure 7F, Supporting Information). These data strongly suggest that HIF is a transcriptional factor that regulates OTUD6B expression under hypoxic condition in HCC cells. Thus, we propose that HIF‐1α is stabilized and accumulated in HCC cells at the early stage upon hypoxia. After 12 h of hypoxia, OTUD6B, as a target of HIF‐1α, is transcriptionally upregulated which leads to pVHL stabilization and HIF‐1α destabilization (Figure 7H).

### 2.8 Conclusion and Discussion

Here we reveal a previously unrecognized negative feedback loop among OTUD6B, pVHL, and HIF‐1α, which regulates HCC metastasis under hypoxia and is important for developing novel therapeutic approaches targeting hypoxic microenvironment in HCC (Figure 7H). Overall, under normoxia, HIF‐1α is hydroxylated and then ubiquitylated for quick degradation. Under hypoxia, HIF‐1α becomes stable due to decreased hydroxylation and ubiquitylation. *OTUD6B* is upregulated by accumulated HIF through transcription. The OTUD6B protein binds to pVHL and elongin B directly, promoting the formation of more CBC^VHL^ ligase complex. This process protects pVHL from proteasomal degradation. Then pVHL recognizes and degrades hydroxylated or SUMOylated HIF‐1α under hypoxia. In this proposed model, OTUD6B is a direct transcriptional target of HIF‐1α, and HIF‐1α is a direct ubiquitylation and degradation target of OTUD6B‐CBC^VHL^ complex. This feedback loop might play a critical role in the maintenance of homeostasis under hypoxia.

Hypoxia is particularly frequent in HCC due to its rapid growing nature. pVHL/HIF‐α axis is the main regulator of hypoxia signaling. pVHL has been documented to be an important tumor suppressor for many malignant tumors. However, to date, the regulatory mechanism of pVHL in HCC cells still remains unclear. Previous studies have shown that the stability of pVHL is regulated by UPS ^[^ ^24^, ^25^ ^]^ and several ubiquitylating enzymes participate in the pVHL stability control. E2‐EPF UCP, one of member of E2 enzyme family, has been demonstrated to interact with pVHL and catalyze an E3‐independent ubiquitylation of pVHL, followed by the destruction of pVHL via 26S proteasome.^[^ ^24^, ^28^, ^29^, ^30^ ^]^ WSB1, a newly identified E3 ligase for pVHL, was shown to promote cancer invasion and metastasis through its effect on pVHL.^[^ ^25^ ^]^ In the present study, we discovered that OTUD6B, a member of OTU DUBs, is able to remove the ubiquitin conjugation of pVHL and stabilize pVHL in HCC cells. So far as we know, OTUD6B is the first identified DUB that harbors the stabilizing ability on pVHL in HCC cells.

DUBs regulate virtually all ubiquitin‐dependent processes by specifically cleaving the isopeptide or peptidic bond and removing ubiquitin from the targeted proteins.^[^ ^27^ ^]^ More than 100 DUBs are predicted to be active in human cells and many of them have been implicated in human diseases including cancer.^[^ ^46^ ^]^ Among them, the OTU subfamily has emerged as regulators of predominant signaling cascades in part due to their specificity for recognition of polyubiquitin chain linkage.^[^ ^46^ ^]^ For example, OTUB1 inhibits noncanonical DNA damage‐dependent ubiquitylation.^[^ ^38^ ^]^ OTUD3 suppresses tumorigenesis by stabilizing tumor suppressor phosphate and tension homology and then suppressing PI3K/Akt pathway.^[^ ^47^ ^]^ OTUD7B promotes NF‐κB signaling through deubiquitylating TRAF3.^[^ ^48^ ^]^ OTULIN, the linear ubiquitin chain‐specific DUB, is essential for the balance of TNF‐associated systemic inflammation in human and mice.^[^ ^49^ ^]^ Compared with these members, less is known about the function and mechanism of OTUD6B. Recent studies found that biallelic pathogenic variants in OTUD6B in 12 individuals from 6 independent families with an intellectual disability syndrome associated with seizures and dysmorphic features.^[^ ^50^, ^51^ ^]^ Homozygous *OTUD6B* knockout mice were dead at the birth day, smaller in size, and had congenital heart defects.^[^ ^50^ ^]^ Here, we found that OTUD6B knockdown dramatically enhances migration features of human HCC cells in vitro and in vivo. And the analysis of human HCC samples showed low OTUD6B level is correlated with poor survival and high recurrence rate of HCC patients. This study provides direct evidence to identify OTUD6B as a tumor suppressor for HCC metastasis, and also couple OTUD6B to the cancer‐related pVHL‐HIF signaling pathway. These findings will expand our understanding on the physiological and pathophysiological functions of the OTU deubiquitylases.

Human *OTUD6B* maps to chromosome 8q21.3 and consists of seven exons that encode a 314‐amino acid protein. The predicted protein contains several coiled‐coil motifs in the N‐terminal part and a conserved OTU domain in the C‐terminal part, which is associated with cysteine protease activity.^[^ ^50^ ^]^ The predicted catalytic residues that function together at the center of the enzyme's active site are Asp185, Cys188, and His307.^[^ ^50^ ^]^ Surprisingly, our results showed that OTUD6B‐mediated pVHL deubiqutylation is independent of the catalytic activity of OTU domain in cells. Purified OTUD6B protein lost the ability of deubiquitylating pVHL in ubiquitylation assay in vitro, in line with previous findings that purified OTUD6B is not able to cleave the ubiquitin chain in cell‐free system.^[^ ^46^ ^]^ Interestingly, we found that the N‐terminal part of OTUD6B is essential for its effect on pVHL. We showed that the N‐terminal part of OTUD6B was able to interact with both pVHL and elongin B. It seems that OTUD6B can be incorporated into the Cullin 2‐elongin B/C‐pVHL complex and function as a regulatory component. This is a really interesting new mode that a putative DUB works together with a Cullin‐RING E3 ligase complex. It is worthy to further explore the structural basis of pVHL recognition by the N‐terminal part of OTUD6B for understanding how OTUD6B and pVHL interact with each other.

Additionally, we observed that OTUD6B is able to bind to the β‐domain of pVHL and elongin B, a subunit of CBC^VHL^ complex, respectively and enhances the interaction between pVHL and elongin B/C complex, which has been suggested to protect pVHL from proteasomal degradation.^[^ ^41^, ^42^ ^]^ We suggest that OTUD6B might stabilize pVHL through increasing its association with elongin B/C and decreasing the interaction between pVHL and the known ubiquitylating enzymes. Considering a half of the tumor‐driven pVHL mutations harbor in α domain of pVHL, which interferes with its ability to bind elongin C and leads to the loss of pVHL protein stability,^[^ ^14^, ^41^, ^42^ ^]^ we propose that OTUD6B overexpression resists HIF‐1α accumulation in tumor cells with pVHL α domain mutations.

The cellular response to hypoxia is a stress response that permits cells to effectively counteract stresses and survive. However, persistent activation of HIF pathway can indeed contribute to tumor development. The equilibrium of HIF signaling is essential for maintaining oxygen homeostasis in cells. Our previous study has demonstrated the presence of positive feedback loop between HIF‐1α and SENP1 deSUMOylase which contributes to the maintenance of HCC stemness and tumorigenesis under hypoxia.^[^ ^8^ ^]^ Here, intriguingly we found that OTUD6B is induced by hypoxia as a direct target of HIF in HCC cells. We revealed a previously unrecognized negative feedback loop between HIF‐1α and OTUD6B. Overall, hypoxia‐induced expression of OTUD6B increased stability of pVHL, which recognizes hydroxylated or SUMOylated HIF‐1α under hypoxia,^[^ ^52^, ^53^ ^]^ and leads to rapid polyubiquitylation and proteasomal degradation of HIF‐1α and suppressed tumor metastasis. This is valuable for understanding the regulatory mechanism of HIF pathway and exploring novel therapeutic approach target tumor environment.

## 3 Experimental Section

##### Cell Culture and Cloning Procedures

All cell lines were maintained in Dulbecco's modified Eagle's medium containing 1% penicillin and streptomycin, supplemented with 10% fetal bovine serum (FBS). 1% O_2_ was generated by flushing a 94% N2/5% CO_2_ mixture into the incubator. All expression plasmids are shown in the Supporting Information.

##### Lentivirus Packaging and Infection

To generate the lentivirus shRNA constructs against human OTUD6B, HIF‐1/2α, or pVHL, shRNA sequences were cloned into the pLKO.1‐puro vector. The shRNA sequences are reported in Table S5 (Supporting Information). pLKO.1, pVSVG, pREV, and pGAG were cotransfected into HEK293T cells for 24 h, and cell culture media was collected. The full‐length of indicated genes were cloned into the pCDH‐puro vector. pCDH, pSPAX.2, and pMD.2 G were cotransfected into HEK293T cells for 24 h, and cell culture media was collected. The viruses were used to infect cells in the presence of polybrene. Forty‐eight hours later, HCC cells were cultured in medium containing puromycin for the selection of stable clones. The clones stably knocking down or overexpressing OTUD6B or pVHL were identified and verified by western blotting.

##### Cell Migration Assays

Cell migration assays were performed in 24 well transwell plate with 8 mm polyethylene terephthalate membrane filters (Corning) separating the lower and upper culture chambers. In brief, cells were plated in the upper chamber at 5 × 10^4^ cells per well in serum‐free DMEM medium. The bottom chamber contained DMEM medium with 10% FBS. Cells were allowed to migrate for 24 h in a humidified chamber at 37 °C with 5% CO_2_. After the incubation period, the filter was removed and nonmigrant cells on the upper side of the filter were detached using a cotton swab. Filters were fixed with 4% formaldehyde for 15 min and cells located in the lower filter were stained with 0.1% crystal violet for 20 min and photographed.

##### Animal Experiments

Animal care and experiments were performed in strict accordance with the “Guide for the Care and Use of Laboratory Animals” and the “Principles for the Utilization and Care of Vertebrate Animals” and were approved by the Experimental Animal Ethical Committee at Beijing Institute of Lifeomics. BALB/c nude mice (6‐week old, 18.0 ± 2.0 g) were obtained from Beijing Vital River Laboratory and were randomly divided into indicated groups. The mice in the groups were injected with the indicated cells expressing luciferase through lateral tail vein. All animals were killed 8 weeks after injection, and the liver and lung tissues were isolated for IVIS Lumina Series III imaging analysis and paraffin‐embedded sections to check formation of primary tumor and metastasis by histology hematoxylin and eosin (H&E) staining.

##### Cohort and Immunohistochemistry

Tumor tissue microarrays (HlivH180su14), purchased from Shanghai Biotech Company, contain 90 pairs of hepatocellular carcinomas together with matched adjacent normal hepatocellular tissue and follow‐up (range 0–120 months). This process had fully informed consent of the patients. Immunohistochemistry was performed by using the avidin–biotin complex method (Vector Laboratories), including heat‐induced antigen‐retrieval procedures. Incubation with antibodies against OTUD6B (1:150; NBP1‐85652, Novas), pVHL (1:150; NB100‐485, Novas) was carried out at 4 °C for 12 h. All staining was assessed by a quantitative imaging method; the percentage of immunostaining and the staining intensity were recorded. An H‐score was calculated using the following formula: H‐score = Σ(PI × *I*) = (percentage of cells of weak intensity × 1) + (percentage of cells of moderate intensity × 2) + (percentage of cells of strong intensity × 3). PI indicates the percentage of positive cells versus all cells, and I represents the staining intensity.

##### Cell Transfections, Immunoprecipitation, and Immunoblotting

Cells were transfected with indicated plasmids using Lipofectamine 2000 (Invitrogen) reagent according to the manufacturer's protocol. For immunoprecipitation assays, cells were lysed with NP40 lysis buffer (50 × 10^−3^ m Tris–HCl, pH 8.0, 150 × 10^−3^ m NaCl, 1% NP40, 0.5% deoxycholate) supplemented with protease‐inhibitor cocktail (Biotool). Immunoprecipitations were performed using the indicated primary antibody and protein A/G agarose beads (Santa Cruz) at 4 °C. The immunocomplexes were then washed with 200 µL phosphate buffered saline (PBS) for twice. Both lysates and immunoprecipitates were examined using the indicated primary antibodies followed by detection with the related secondary antibody and the Western Bright ECL chemiluminescent Detection Reagent (Advansta). All the primary antibodies were listed in Table S6 (Supporting Information).

##### Ubiquitylation Assay in Cells

OTUD6B‐dependent regulation of (covalent) pVHL or HIF‐1α ubiquitination were examined using Ubiquitylation assay in cells as described previously.^[^ ^54^ ^]^ HA‐Ub or His‐Ub was cotransfected together with flag (myc)‐pVHL. Cells were treated with MG132 for 8 h before collection. Then HA‐Ub was immunoprecipitated with anti‐HA antibody, while His‐Ub was pulled down using Ni‐NTA, and immunoblotted with anti‐flag or myc antibody.

##### Glutathione S‐transferase (GST) Pull‐Down Assay

Indicated cDNA was cloned into a pGEX‐4T‐2 vector with an N‐terminal GST‐tag and purified from the *Escherichia coli* strain BL21 (Invitrogen) using GST Agarose beads. Bacterial‐expressed GST and GST‐OTUD6B protein bound to glutathione‐sepharose 4B beads (from GE) was incubated with pVHL expressed in HEK293T cells for overnight at 4 °C. Then the beads were washed with PBS four times, followed by western blotting.

##### In Vitro Ubiquitylation Assay

His‐WSB1 and GST‐pVHL were expressed in HEK293T and purified. Indicated proteins were pretreated at 30 °C for 30 min. Afterward, 0.7 µg of E1, 0.9 µg of UbcH5c, 12 µg of HA‐ubiquitin, 0.7 µg of His‐WSB1, and 1.6 µg GST‐VHL were resolved in ubiquitylation assay buffer (5 × 10^−3^ m Mg‐adenosine triphosphate, 5 × 10^−3^ m ethylene diamine tetraacetic acid (EDTA), 1 × 10^−3^ m DTT, and 10 U mL^−1^ Inorganic pyrophosphatase were included). The reactions were stopped by the addition of dodecyl sulfate,sodium salt (SDS)‐polyacrylamide gel electrophoresis (PAGE) sample buffer. The reaction products were resolved by SDS–PAGE gel and probed with the indicated antibodies.

##### Realtime (RT)‐quantitative Polymerase Chain Reaction

Total RNA was extracted with TRIzol (Invitrogen) and precipitated in ethanol. Total RNA (1 µg) was reverse transcribed into cDNA using SuperScript III First‐Strand Synthesis SuperMix (Invitrogen, 11752‐50). A 20 µL volume reaction consisted of 1 µL reverse transcription product and 250 × 10^−9^ m of each primer. The primers used for the indicated gene products are described in Table S7 (Supporting Information).

##### Chromatin Immunoprecipitation Assay^[^ ^8^ ^]^

HCC cells were crosslinked with formaldehyde, lysed with SDS buffer, and sonicated. Sheared DNA was precleared with salmon sperm DNA/protein A agarose slurry (Merck Millipore) and immunoprecipitated with HIF‐1α or HIF‐2α antibody and IgG (Santa Cruz). Agarose beads were incubated with antibody/protein/DNA complex and washed with low‐salt buffer, high‐salt buffer, and LiCl wash buffer according to manufacturer's protocol (Millipore). DNA was eluted in 1% SDS/0.1 m NaHCO_3_, de‐crosslinked with NaCl (0.2 m), and extracted by phenol–chloroform.

##### Gel Filtration Chromatography Analysis

Liver cancer cells with NTC or OTUD6B knockdown (three 10 cm dishes for each sample per gel filtration experiment) were treated with MG132 for 8 h, and then washed with phosphate‐buffered saline, lysed in 0.5 mL of 3‐[(3‐Cholamidopropyl)dimethylammonio]‐1‐propanesulfonate (CHAPS) lysis buffer (25 × 10^−3^ m *N*‐2‐hydroxyethylpiperazine‐*N*‐ethane‐sulphonicacid at pH 7.4, 150 × 10^−3^ m NaCl, 1 × 10^−3^ m EDTA, and 0.3% CHAPS) containing protease inhibitors (Complete Mini, Roche) and phosphatase inhibitors (phosphatase inhibitor cocktail set I and II, Calbiochem), and filtered through a 0.45 µm syringe filter. Total protein concentration was then adjusted to 8 mg mL^−1^ with CHAPS buffer and 500 µL of the lysate was loaded onto a Superdex 200 10/300 GL column (GE Lifesciences Cat. No. 17‐5175‐01). Chromatography was performed on the AKTA‐FPLC (GE Lifesciences Cat. No. 18‐1900‐26) with CHAPS buffer. One column volume of eluates was fractionated with 500 mL in each fraction, at the elution speed of 0.5 mL min^−1^. 30 μL aliquots of each fraction were loaded onto SDS–PAGE gels and detected with indicated antibodies.

##### RNA‐Sequencing Analysis

The RNA‐seq library was prepared for sequencing using standard Illumina protocols. Total RNA samples from OTUD6B knockdown and NTC MHCC‐LM3 cells were isolated using TRIzol reagent (Invitrogen) and treated with RNase‐free DNase I (New England Biolabs, MA, USA), to remove any contaminating genomic DNA. Library construction and sequencing were performed by Beijing Genomics Institution. For the data analysis, basecalls are performed using the internal assembler and variant caller. Clean reads were aligned to the genome using STAR (v2.5.1b) and HTSeq v0.6.0 was used to count the read numbers mapped to each gene. Differential expression was determined using the edgeR package and the significance of the differential expression of genes was defined by the bioinformatics service according to the combination of the absolute value of log2‐fold change ≥ 1 and *P* value ≤ 0.01. GO, and pathway annotation and enrichment analyses were based on the Gene Ontology Database (https://www.geneontology.org/), and KEGG pathway database (https://www.genome.jp/kegg/), respectively. The software Cluster and Java Treeview were used for hierarchical cluster analysis of gene expression patterns. The original sequence data have been submitted to the database of the NCBI Sequence Read Archive (https://trace.ncbi.nlm.nih.gov/traces/sra) under the accession number PRJNA555489.

##### Statistical Analysis

All the statistical analyses were performed by the statistical package for social science (SPSS). Student's *t* test, two‐way ANOVA test, Chi Square or Mann–Whitney tests were used. *P* values less than 0.05 were considered significant.

## Conflict of Interest

The authors declare no conflict of interest.

## Supporting information

Supporting Information
