# Supplementary material for: Deubiquitylase OTUD6B Governs pVHL Stability in an Enzyme‐Independent Manner and Suppresses Hepatocellular Carcinoma Metastasis
Source: Adv Sci (Weinh). 2020 Mar 8;7(8):1902040. doi: 10.1002/advs.201902040 (PMC7175249; doi:10.1002/advs.201902040)
Supplement: Supplementary file 1 — Supporting Information [file ADVS-7-1902040-s001.pdf]

Copyright WILEY-VCH Verlag GmbH & Co. KGaA, 69469 Weinheim, Germany, 2020.

ADVANCED SCIENCE
Open Access

Supporting Information

for *Adv. Sci.,* DOI: 10.1002/advs.201902040

Deubiquitylase OTUD6B Governs pVHL Stability in an Enzyme-Independent Manner and Suppresses Hepatocellular Carcinoma Metastasis

Xinxin Liu, Xiaoli Zhang, Zhiqiang Peng, Chunnan Li, Ze Wang, Chanjuan Wang, Zhikang Deng, Bo Wu, Yu Cui, Zhanxin Wang, Chun-Ping Cui, Min Zheng,* and Lingqiang Zhang**

Supporting Information

Deubiquitylase OTUD6B governs pVHL stability in an enzyme-independent manner and suppresses hepatocellular carcinoma metastasis

Xinxin Liu[#], Xiaoli Zhang[#], Zhiqiang Peng[#], Chunnan Li, Ze Wang, Chanjuan Wang, Zhikang Deng, Zhanxin Wang, Bo Wu, Yu Cui, Chun-Ping Cui[*], Min Zheng[*], Lingqiang Zhang[*]

[#]These authors contributed equally to this work.

**Correspondence should be addressed to L.Z. (e-mail: zhanglq@nic.bmi.ac.cn), M.Z. (e-mail: minzheng@zju.edu.cn) and C.P.C. (e-mail: cui_chunping2000@aliyun.com)*

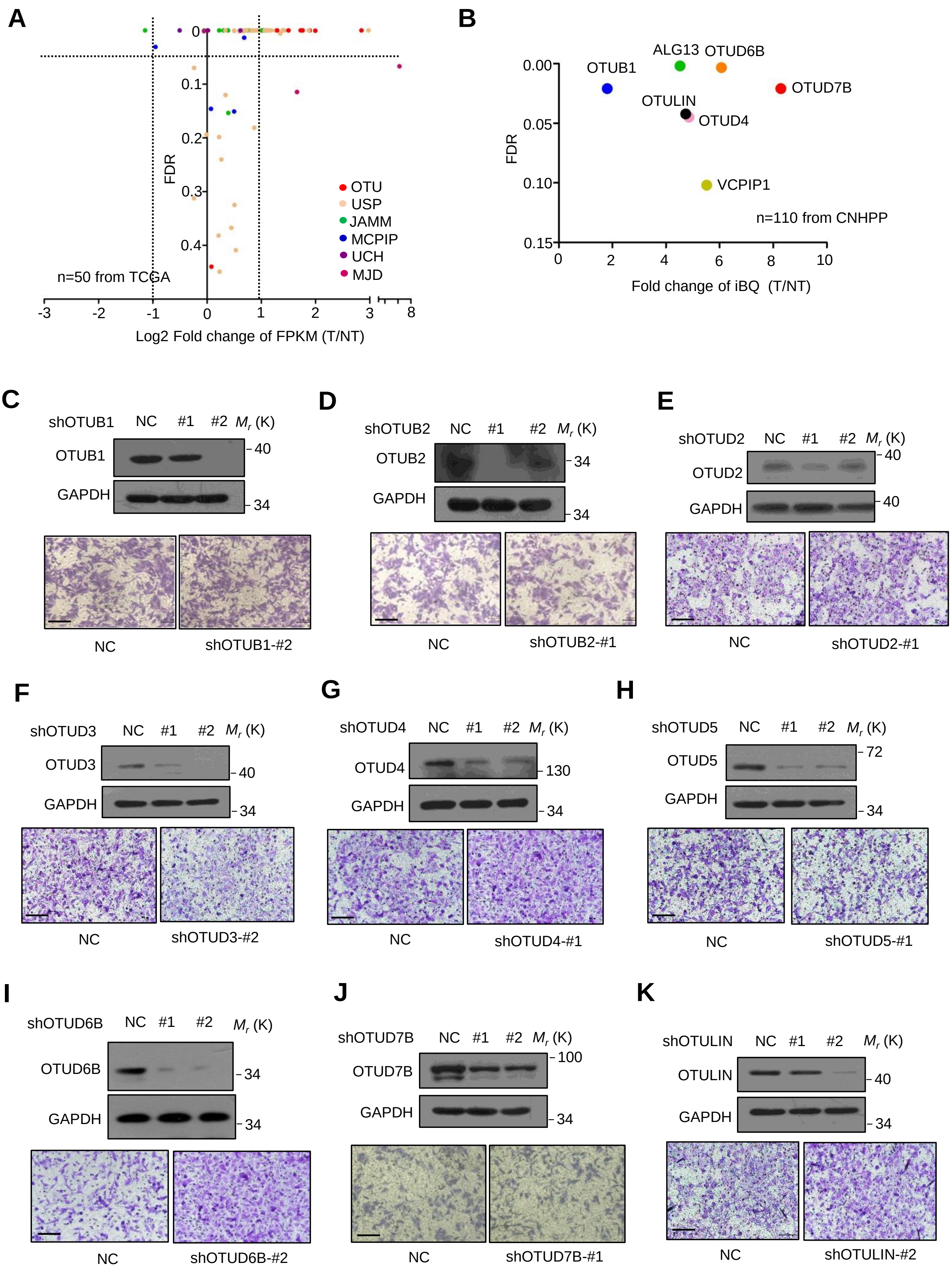
A
B
C
D
E
F
G
H
I
J
K
n=50 from TCGA
Log2 Fold change of FPKM (T/NT)
FDR
OTU
USP
JAMM
MCPIP
UCH
MJD
n=110 from CNHPP
Fold change of iBQ (T/NT)
ALG13
OTUD6B
OTUB1
OTUD7B
OTULIN
OTUD4
VCPIP1
shOTUB1 NC #1 #2 M_r (K)
OTUB1
GAPDH
NC
shOTUB1-#2
shOTUB2 NC #1 #2 M_r (K)
OTUB2
GAPDH
NC
shOTUB2-#1
shOTUD2 NC #1 #2 M_r (K)
OTUD2
GAPDH
NC
shOTUD2-#1
shOTUD3 NC #1 #2 M_r (K)
OTUD3
GAPDH
NC
shOTUD3-#2
shOTUD4 NC #1 #2 M_r (K)
OTUD4
GAPDH
NC
shOTUD4-#1
shOTUD5 NC #1 #2 M_r (K)
OTUD5
GAPDH
NC
shOTUD5-#1
shOTUD6B NC #1 #2 M_r (K)
OTUD6B
GAPDH
NC
shOTUD6B-#2
shOTUD7B NC #1 #2 M_r (K)
OTUD7B
GAPDH
NC
shOTUD7B-#1
shOTULIN NC #1 #2 M_r (K)
OTULIN
GAPDH
NC
shOTULIN-#2

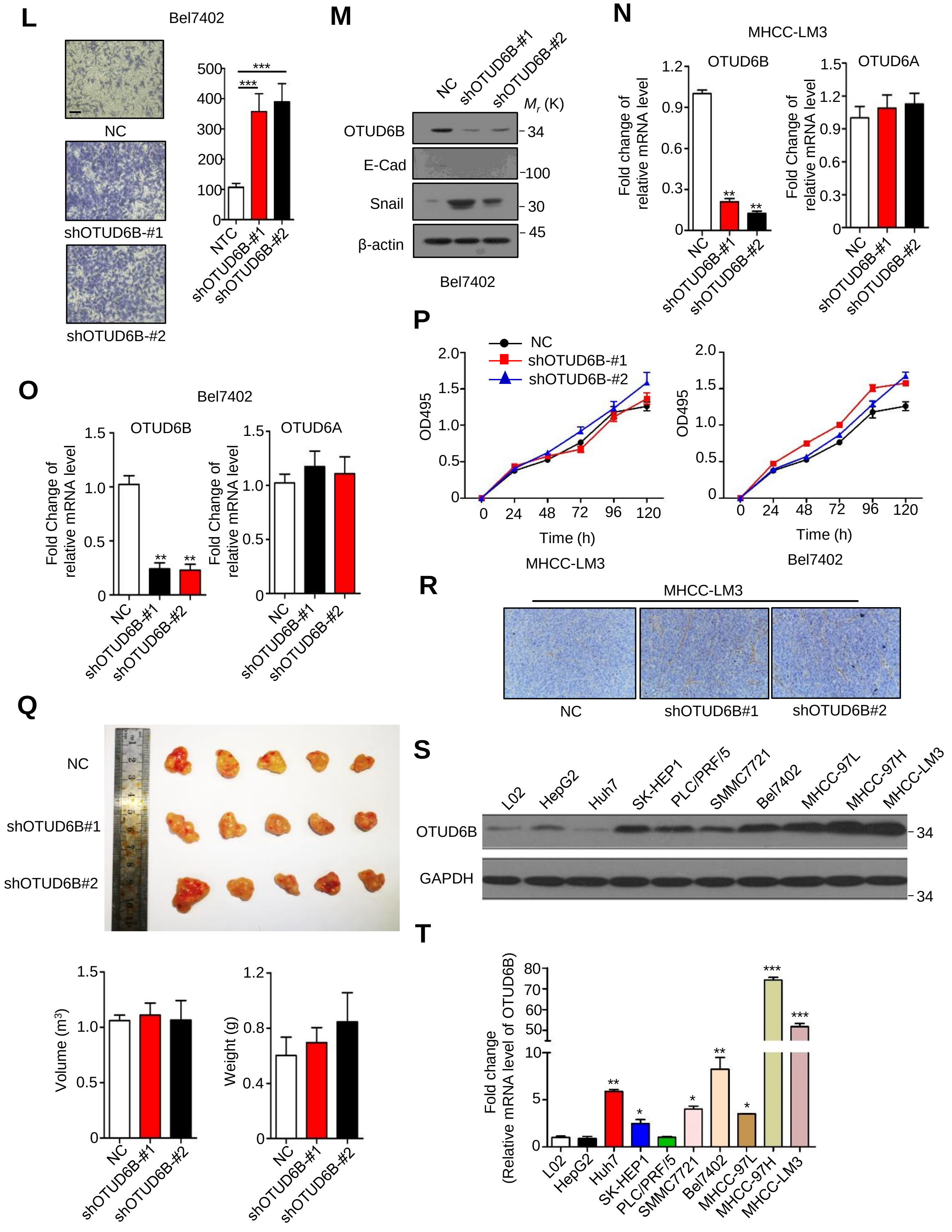

L
Bel7402
NC
shOTUD6B-#1
shOTUD6B-#2
NTC
***
M
NC
shOTUD6B-#1
shOTUD6B-#2
M_r (K)
OTUD6B
E-Cad
Snail
β-actin
Bel7402
N
MHCC-LM3
OTUD6B
OTUD6A
Fold change of relative mRNA level
Fold Change of relative mRNA level
O
Bel7402
P
NC
OD495
Time (h)
MHCC-LM3
R
Q
shOTUD6B#1
shOTUD6B#2
Volume (m³)
Weight (g)
S
L02
HepG2
Huh7
SK-HEP1
PLC/PRF/5
SMMC7721
MHCC-97L
MHCC-97H
GAPDH
T
Fold change (Relative mRNA level of OTUD6B)

Supplemental Figure 1. OTUD6B suppresses HCC metastasis. (A) Scatter plot of 98 members of DUBs mRNA expression in human HCC tissues from TCGA data of National Cancer Institute, USA. The X-axis represents the value of log2 (fold change of DUBs PKFM in tumor tissues (T) / non-tumor liver tissue (NT) in HCC patients, and the Y-axis represents the value of false positive rate (FDR). **(B)** Scatter plot of the protein levels of OTU members in human HCC tissues from CNHPP data. The X-axis represents the fold change of OTUs iBQ in tumor tissues (T) / non-tumor liver tissue (NT) in HCC patients, and the Y-axis represents the value of false positive rate (FDR). **(C-K)** Transwell assays were used to test the effect of depletion of OTU family DUB members, including OTUB1 (C), OTUB2 (D), OTUD2 (D), OTUD3 (E), OTUD4 (F), OTUD5 (G), OTUD6B (H), OTUD7B (I) or OTULIN (K) on cell migration in MHCC-LM3 cells. **(L)** Transwell assays were used to examine the effect of depletion of OTUD6B on cell migration in Bel7402 cell. Results are shown as mean ± s.d. n=3 independent experiments. **, $P < 0.01$, ***, $P < 0.001$, student *t* test. **(M)** Immunoblot assays were conducted with indicated antibodies in HCC cells (Bel7402) with OTUD6B stable knockdown. **(N, O)** Real time Q-PCR assays were performed to test the mRNA level of OTUD6B and OTUD6A in MHCC-LM3 (N) and Bel7402 (O) cells. Results are shown as mean ± s.d. n=3 independent experiments. **, $P < 0.01$, student *t* test. **(P)** MTS assays were used to examine cell proliferation of HCC cells with OTUD6B knockdown. **(Q)** 5×10^6 MHCC-LM3 cells with indicated shRNA were injected into nude mice subcutaneously. The mice were euthanized 6 weeks later by a cervical dislocation. Three dimensions of tumors were measured with caliper and tumor volume was calculated with formula length x width x depth x 0.52 (mm^3). The tumor weight was calculated and shown. Results are shown as mean ± s.d. n=5 independent experiments. **(R)** Immunohistochemistry (IHC) was performed with anti-CD31 antibody on tumor tissues. **(S, T)** The protein (S) and mRNA (T) level of

OTUD6B in a panel of HCC cell lines. Results are shown as mean ± s.d. n=3 independent experiments. *, $P < 0.05$, **, $P < 0.01$, ***, $P < 0.001$, student *t* test.

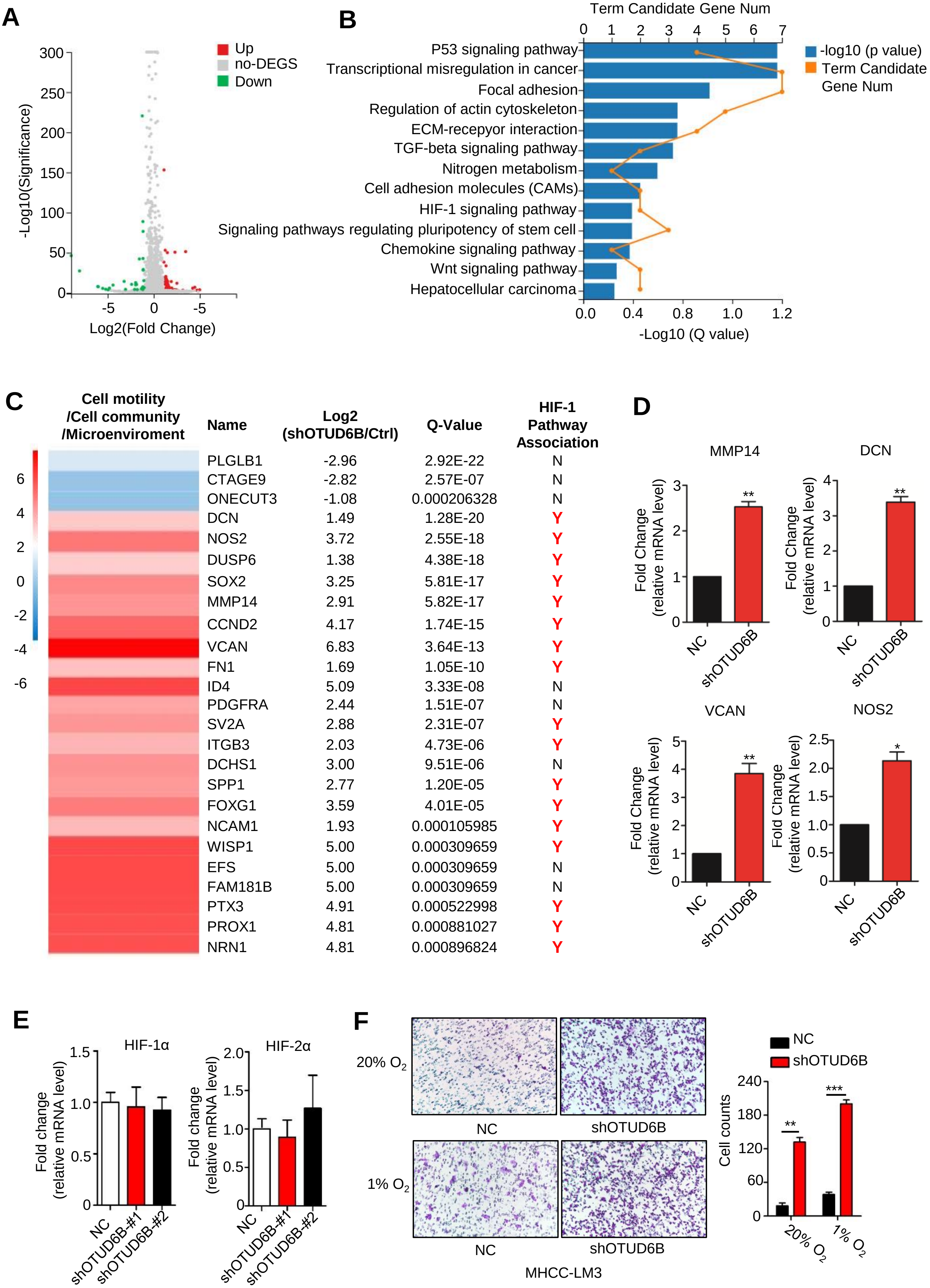
A
Up
no-DEGS
Down
-Log10(Significance)
Log2(Fold Change)
B
Term Candidate Gene Num
P53 signaling pathway
Transcriptional misregulation in cancer
Focal adhesion
Regulation of actin cytoskeleton
ECM-recepyor interaction
TGF-beta signaling pathway
Nitrogen metabolism
Cell adhesion molecules (CAMs)
HIF-1 signaling pathway
Signaling pathways regulating pluripotency of stem cell
Chemokine signaling pathway
Wnt signaling pathway
Hepatocellular carcinoma
-log10 (p value)
Term Candidate Gene Num
-Log10 (Q value)
C
Cell motility /Cell community /Microenviroment
Name | Log2 (shOTUD6B/Ctrl) | Q-Value | HIF-1 Pathway Association
PLGLB1 -2.96 2.92E-22 N
CTAGE9 -2.82 2.57E-07 N
ONECUT3 -1.08 0.000206328 N
DCN 1.49 1.28E-20 Y
NOS2 3.72 2.55E-18 Y
DUSP6 1.38 4.38E-18 Y
SOX2 3.25 5.81E-17 Y
MMP14 2.91 5.82E-17 Y
CCND2 4.17 1.74E-15 Y
VCAN 6.83 3.64E-13 Y
FN1 1.69 1.05E-10 Y
ID4 5.09 3.33E-08 N
PDGFRA 2.44 1.51E-07 N
SV2A 2.88 2.31E-07 Y
ITGB3 2.03 4.73E-06 Y
DCHS1 3.00 9.51E-06 N
SPP1 2.77 1.20E-05 Y
FOXG1 3.59 4.01E-05 Y
NCAM1 1.93 0.000105985 Y
WISP1 5.00 0.000309659 Y
EFS 5.00 0.000309659 N
FAM181B 5.00 0.000309659 N
PTX3 4.91 0.000522998 Y
PROX1 4.81 0.000881027 Y
NRN1 4.81 0.000896824 Y
D
MMP14
DCN
VCAN
NOS2
Fold Change (relative mRNA level)
NC
shOTUD6B
E
HIF-1α
HIF-2α
Fold change (relative mRNA level)
NC
shOTUD6B-#1
shOTUD6B-#2
F
20% O2
1% O2
NC
shOTUD6B
MHCC-LM3
Cell counts
NC
shOTUD6B

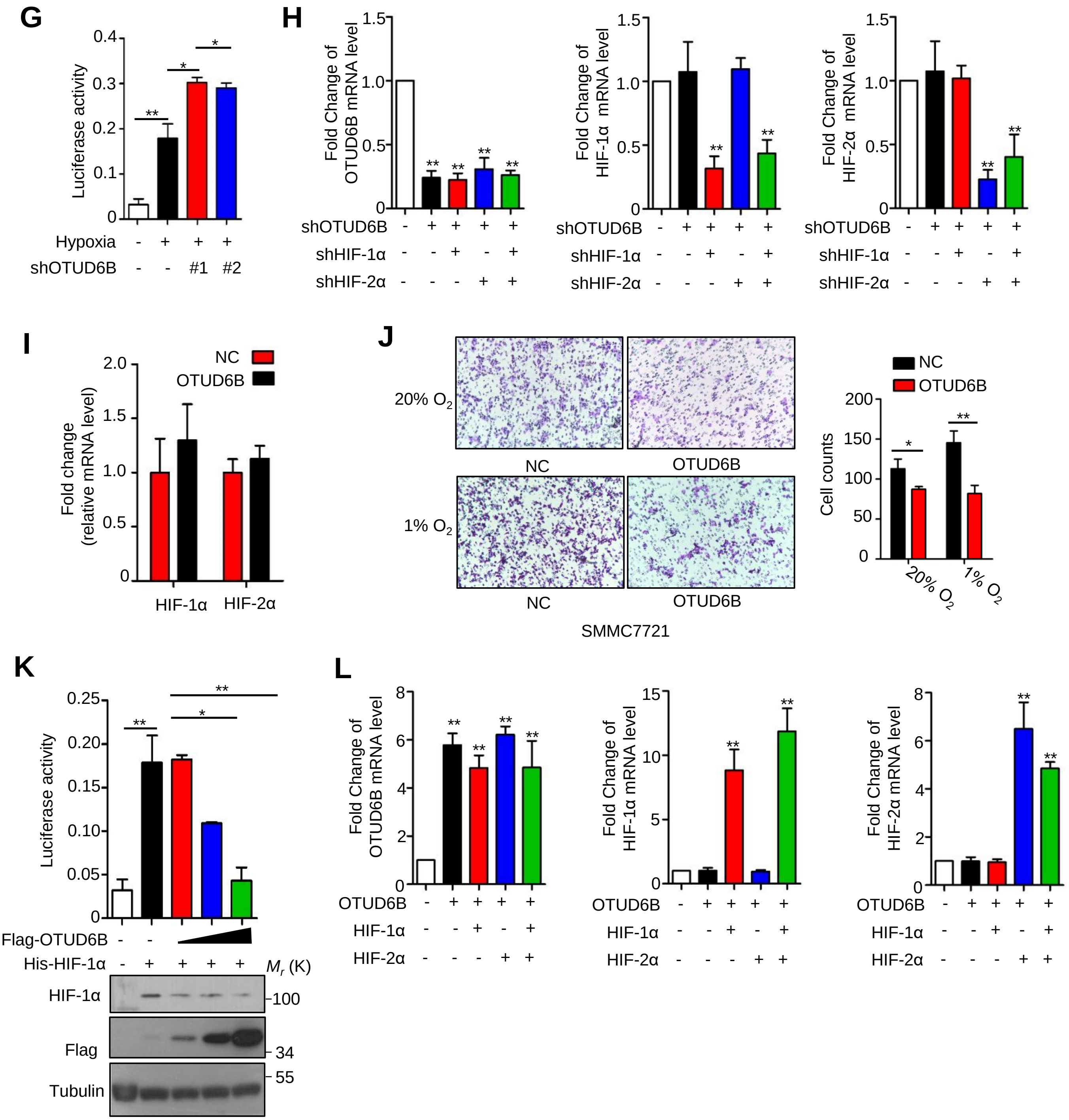
G
Luciferase activity
Hypoxia - + + +
shOTUD6B - - #1 #2
H
Fold Change of OTUD6B mRNA level
Fold Change of HIF-1α mRNA level
Fold Change of HIF-2α mRNA level
shOTUD6B - + + + +
shHIF-1α - - + - +
shHIF-2α - - - + +
I
NC
OTUD6B
Fold change (relative mRNA level)
HIF-1α
HIF-2α
J
20% O2
1% O2
NC
OTUD6B
SMMC7721
Cell counts
20% O2
1% O2
K
Luciferase activity
Flag-OTUD6B
His-HIF-1α - + + + +
Mr (K)
HIF-1α
Flag
Tubulin
100
34
55
L
Fold Change of OTUD6B mRNA level
Fold Change of HIF-1α mRNA level
Fold Change of HIF-2α mRNA level
OTUD6B - + + + +
HIF-1α - - + - +
HIF-2α - - - + +

Supplemental Figure 2. OTUD6B negatively regulates HIF pathway. (A) Identification of the different expression genes (DEGs) was graphed in volcano plot. **(B)** Gene Ontology (GO) enrichment analysis for biological process, cell component, and molecular function of DEGs. The $-\log_{10}$ (Q value of enrichment) is shown on x axis; the numbers represent the number of associated proteins for each term. **(C)** The selected genes involved in cell motility, cell community and microenvironments are shown as a heatmap. **(D)** Real-time Q-PCR assays were used to confirm the results of RNA-Seq. **(E)** Real time Q-PCR was used to examine the mRNA level of HIF-1α and HIF-2α in MHCC-LM3 cells transfected with indicated shRNA. **(F)** Transwell assays were used to examine the effect of depletion of OTUD6B on cell migration in MHCC-LM3 cell under normoxia and hypoxia. **(G)** Fold change of the relative luciferase activity was examined by luciferase-reporter assay in OTUD6B knockdown and NC HCC cells which were incubated under normoxia or hypoxia for 24 hours. **(H)** Real-time Q-PCR assays were used to determine the expression of indicated genes in MHCC-LM3 cells transfected with indicated shRNA. **(I)** Real time Q-PCR was used to examine the mRNA level of HIF-1α and HIF-2α in SMMC7721 cells with ectopic OTUD6B overexpression. **(J)** Transwell assays were used to examine the effect of ectopic OTUD6B on cell migration in SMMC7721 cell. **(K)** Fold change of the relative luciferase activity was examined by luciferase-reporter assay in HCC cells transfected with indicated constructs. **(L)** Real time Q-PCR assays were used to determine the expression of indicated genes in SMMC7721 cells with stable overexpression of indicated genes. (Results are shown as mean ± s.d. n=3 independent experiments. *, $P < 0.05$, **, $P < 0.01$, ***, $P < 0.001$, student *t* test.)

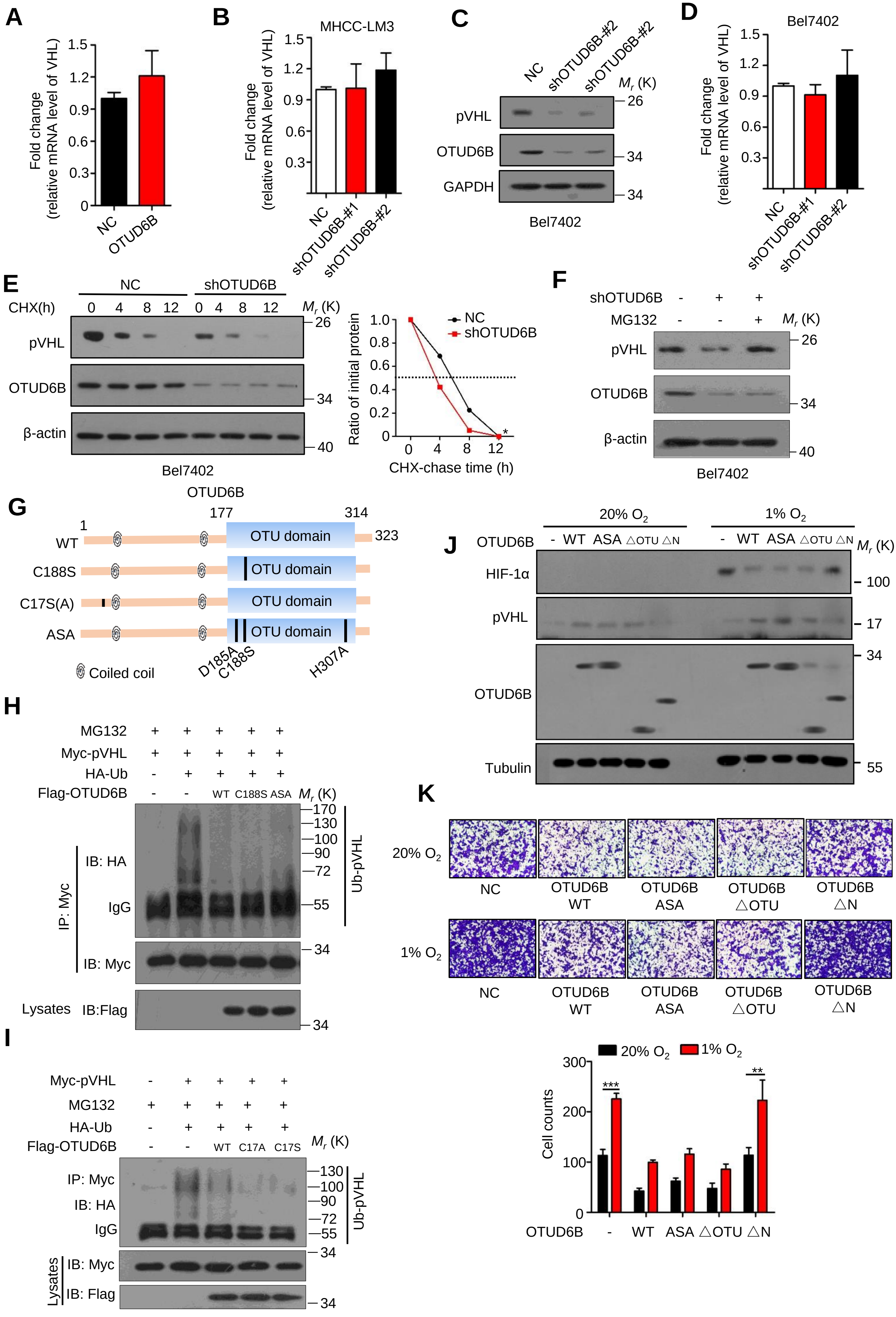
A
B
C
D
MHCC-LM3
Bel7402
Fold change (relative mRNA level of VHL)
NC
OTUD6B
shOTUD6B-#1
shOTUD6B-#2
pVHL
OTUD6B
GAPDH
Mr (K)
E
CHX(h)
shOTUD6B
β-actin
Ratio of initial protein
CHX-chase time (h)
F
MG132
G
WT
C188S
C17S(A)
ASA
OTU domain
Coiled coil
D185A
H307A
H
Myc-pVHL
HA-Ub
Flag-OTUD6B
IP: Myc
IB: HA
IgG
IB: Myc
Lysates
IB:Flag
Ub-pVHL
I
C17A
C17S
J
20% O2
1% O2
HIF-1α
Tubulin
△OTU
△N
K
Cell counts

Supplemental Figure 3. OTUD6B deubiquitylates and stabilizes pVHL in an OTU-independent manner. (A) Real time Q-PCR was used to examine the mRNA level of pVHL in SMMC7721 cells with stable OTUD6B overexpression. **(B)** Real-time Q-PCR assays were used to determine the expression of VHL in MHCC-LM3 cells with OTUD6B depletion. **(C)** Immunoblot assays were conducted with indicated antibodies in HCC cells (Bel7402) with OTUD6B stable knockdown. (D) Real time Q-PCR was used to examine the mRNA level of pVHL in Bel7402 cells with stable OTUD6B knockdown. **(E)** Bel7402 HCC cells were treated with 10μg/ml cycloheximide (CHX), and collected at the indicated times for western blot. Quantification of pVHL levels relative to GAPDH is shown. Results are shown as mean ± s.d. n=3 independent experiments. ∗∗, P <0.01, two-way ANOVA test. **(F)** Immunoblot assays were conducted with indicated antibodies in HCC cells (Bel7402) with OTUD6B stable knockdown. **(G)** Overview of the structures of OTUD6B wild type and different truncates. **(H, I)** HA-Ub was co-transfected together with indicated constructs into HEK293T cells. Cells were treated with MG132 for 8h before collection. Then pVHL was immunoprecipitated with anti-myc antibody and immunoblotted with anti-HA antibody. **(J)** Immunoblot assays were conducted with indicated antibodies in HCC cells (SMMC7721) with stable overexpression of OTUD6B wild type and different truncates. **(K)** Transwell assays were used to examine the effect of OTUD6B wild type and different truncates on cell migration in SMMC7721 cell. Results are shown as mean ± s.d. n=3 independent experiments. **, P < 0.01, ***, P < 0.001, student *t* test.

Figure S4

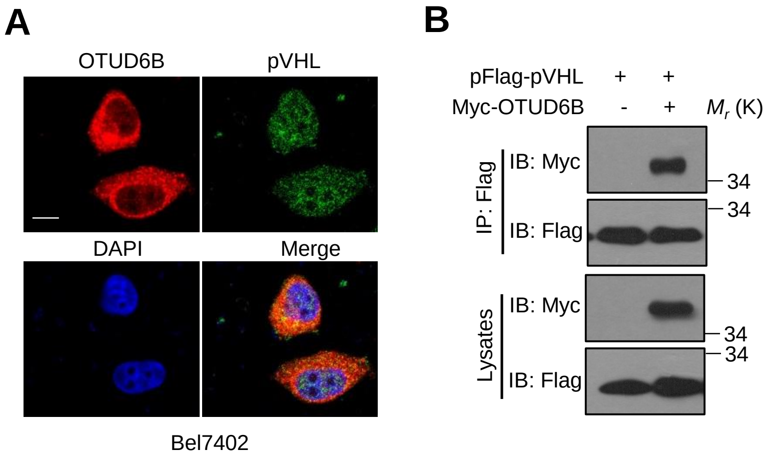
A
OTUD6B
pVHL
DAPI
Merge
Bel7402
B
pFlag-pVHL + +
Myc-OTUD6B - +
M_r (K)
IP: Flag
IB: Myc
34
34
IB: Flag
Lysates
IB: Myc
34
34
IB: Flag

Supplemental Figure 4. OTUD6B interacts with pVHL. (A) Immunofluorescence staining was performed in HCC cells (Bel7402) with anti-OTUD6B and pVHL antibodies to determine the co-localization of these two proteins in HCC cells (scale bar, 10 μm). **(B)** The lysates of HEK293T cells transfected with indicated constructs were subjected to immunoprecipitation with anti-Flag. The immunoprecipitates or the eluates were then blotted.

Figure S5

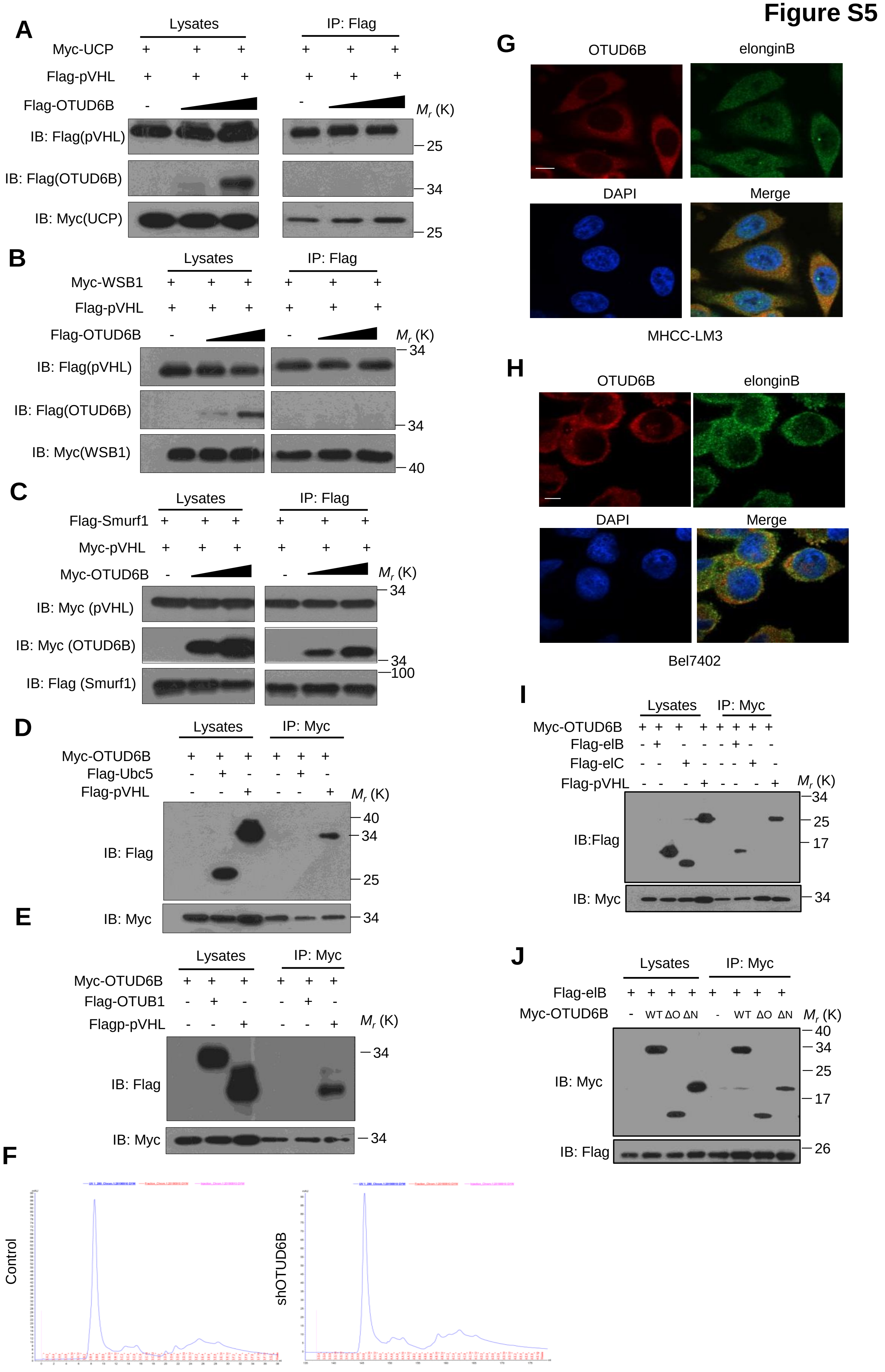
A
Lysates
IP: Flag
Myc-UCP
Flag-pVHL
Flag-OTUD6B
Mr (K)
IB: Flag(pVHL)
IB: Flag(OTUD6B)
IB: Myc(UCP)
25
34
25
B
Myc-WSB1
Flag-pVHL
Flag-OTUD6B
IB: Flag(pVHL)
IB: Flag(OTUD6B)
IB: Myc(WSB1)
34
34
40
C
Flag-Smurf1
Myc-pVHL
Myc-OTUD6B
IB: Myc (pVHL)
IB: Myc (OTUD6B)
IB: Flag (Smurf1)
34
34
100
D
IP: Myc
Myc-OTUD6B
Flag-Ubc5
Flag-pVHL
IB: Flag
IB: Myc
40
34
25
34
E
Myc-OTUD6B
Flag-OTUB1
Flagp-pVHL
IB: Flag
IB: Myc
34
34
F
Control
shOTUD6B
G
OTUD6B
elonginB
DAPI
Merge
MHCC-LM3
H
OTUD6B
elonginB
DAPI
Merge
Bel7402
I
Lysates
IP: Myc
Myc-OTUD6B
Flag-elB
Flag-elC
Flag-pVHL
IB:Flag
IB: Myc
34
25
17
34
J
Lysates
IP: Myc
Flag-elB
Myc-OTUD6B
WT ΔO ΔN
IB: Myc
IB: Flag
40
34
25
17
26

Supplemental Figure 5. OTUD6B interacts with elongin B and enhances the binding of pVHL and elongin C. (A, B, C) Increasing amounts of myc or flag-OTUD6B were co-transfected together with indicated constructs into HEK293T cells and the lysates were subject to immunoprecipitation with anti-myc or flag antibody. The lysates and immunoprecipitates were then blotted. **(D, E)** The lysates of HEK293T cells transfected with indicated constructs were subjected to immunoprecipitation with anti-Myc. The immunoprecipitates or the eluates were then blotted. **(F)** MHCC-LM3 Cells with NC or OTUD6B knockdown were treated with MG132 for 8 hrs, and then lysed in 0.5 ml of CHAPS lysis buffer. 500 µl of the lysate was loaded onto a Superdex 200 10/300 GL column. Chromatography was performed on the AKTA-FPLC with CHAPS buffer. UV280 of different fractions was recorded. **(G, H)** Immunofluorescence staining was performed in HCC cells with anti-OTUD6B and anti-elonginB antibodies to determine the co-localization of these two proteins in MHCC-LM3 (G) and Bel7402 cells (H) (scale bar, 10 µm). **(I, J)** The lysates of HEK293T cells transfected with indicated constructs were subjected to immunoprecipitation with anti-Flag. The immunoprecipitates or the eluates were then blotted.

Figure S6

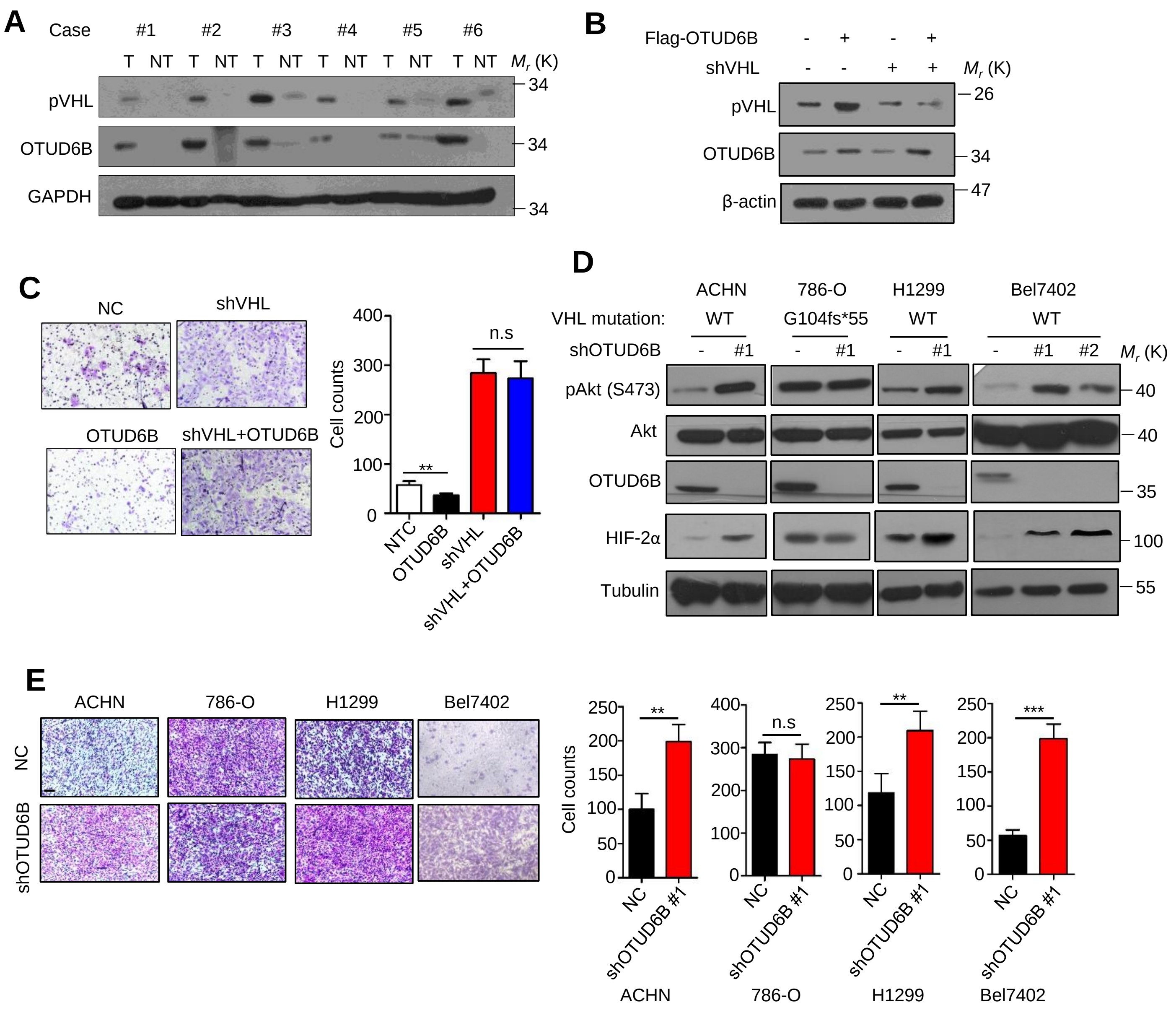

A
Case #1 #2 #3 #4 #5 #6
T NT T NT T NT T NT T NT T NT M_r (K)
pVHL
OTUD6B
GAPDH
34
34
34
B
Flag-OTUD6B - + - +
shVHL - - + +
M_r (K)
pVHL
OTUD6B
β-actin
26
34
47
C
NC
shVHL
OTUD6B
shVHL+OTUD6B
Cell counts
400
300
200
100
0
**
n.s
NTC
OTUD6B
shVHL
shVHL+OTUD6B
D
ACHN 786-O H1299 Bel7402
VHL mutation: WT G104fs*55 WT WT
shOTUD6B - #1 - #1 - #1 - #1 #2
M_r (K)
pAkt (S473)
Akt
OTUD6B
HIF-2α
Tubulin
40
40
35
100
55
E
ACHN 786-O H1299 Bel7402
NC
shOTUD6B
Cell counts
250
200
150
100
50
0
**
400
300
200
100
0
n.s
**
***
NC
shOTUD6B #1
ACHN
786-O
H1299
Bel7402

Supplemental Figure 6. pVHL mediates the inhibitory effect of OTUD6B on HCC cell migration. (A) Immunoblotting of pVHL and OTUD6B in human HCC tumor tissues (T) and the corresponding non-tumor liver tissues (NT). **(B)** Immunoblotting of pVHL and OTUD6B in HCC cells with stable overexpression or knockdown of indicated genes. **(C)** Transwell assays were performed to identify the capacity of migration in HCC cell with stable overexpression or knockdown of indicated genes. **(D)** Immunoblot assays were conducted with indicated antibodies in different types of tumor cells with OTUD6B stable knockdown. **(E)** Tranwell assays were performed to examine the cell migration in indicated types of tumor cells. Results are shown as mean ± s.d. n=3 independent experiments. *, $P < 0.05$, **, $P < 0.01$, ***, $P < 0.001$, student *t* test.

Figure S7

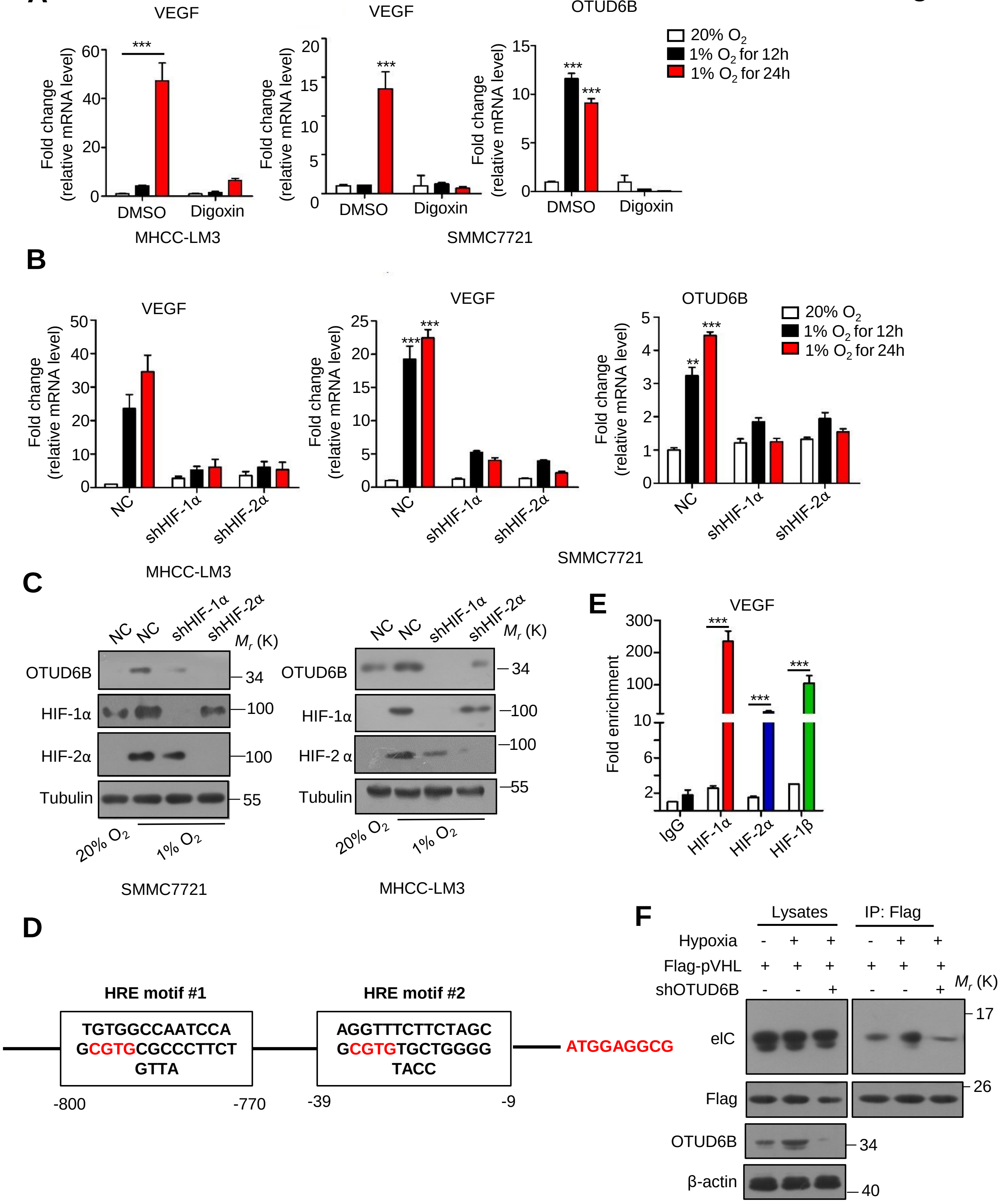

A
VEGF
VEGF
OTUD6B
Fold change (relative mRNA level)
20% O2
1% O2 for 12h
1% O2 for 24h
DMSO
Digoxin
MHCC-LM3
SMMC7721
B
VEGF
VEGF
OTUD6B
NC
shHIF-1α
shHIF-2α
MHCC-LM3
SMMC7721
C
OTUD6B
HIF-1α
HIF-2α
Tubulin
Mr (K)
20% O2
1% O2
SMMC7721
MHCC-LM3
D
HRE motif #1
TGTGGCCAATCCA
GCGTGCGCCCTTCT
GTTA
HRE motif #2
AGGTTTCTTCTAGC
GCGTGTGCTGGGG
TACC
ATGGAGGCG
-800
-770
-39
-9
E
VEGF
Fold enrichment
IgG
HIF-1α
HIF-2α
HIF-1β
F
Lysates
IP: Flag
Hypoxia
Flag-pVHL
shOTUD6B
elC
Flag
OTUD6B
β-actin

Supplemental Figure 7. OTUD6B plays as one of target genes of HIF-1/2α in HCC cells. **(A)** Real time Q-PCR assays were used to determine the expression of indicated genes in HCC cells treated with Digoxin under normoxia or hypoxia. **(B)** Real time Q-PCR assays were used to determine the expression of indicated genes in HCC cells transfected with indicated shRNA under normoxia or hypoxia. **(C)** Immunoblot assays were conducted with indicated antibodies in HCC cells with HIF-1/2α stable knockdown. **(D)** The HRE (hypoxia response element) motifs in OTUD6B promoter were shown. **(E)** Chip assay was used to determine the bind of HIF-1α, HIF-2α and HIF-1β to the HRE sequence in OTUD6B promoter. **(F)** Cell lysates of HCC cells transfected with indicated constructs were subject to immunoprecipitation with anti-flag antibodies. The immunoprecipitates were then blotted with anti-flag antibody. Results are shown as mean ± s.d. n=3 independent experiments. *, $P < 0.05$, **, $P < 0.01$, ***, $P < 0.001$, student *t* test.

Supplemental table 1. The fold change of relative mRNA level of DUBs in HCC tissues and the non-tumor liver tissue.

Name	FC Mean	P value
**USP family**		
USP49	7.869139194	1.09831E-07
USP54	3.989933303	0.000586991
USP21	3.714645962	2.90903E-11
USP35	3.669373819	7.15256E-07
USP46	2.842409008	**3.37269E-08**
USP22	2.838782231	1.05271E-11
USP1	2.684594767	0.000200946
USP31	2.630570425	7.24325E-07
USP27X	2.54864061	0.005831605
USP14	2.414225362	4.62075E-09
USP3	2.399001565	1.36805E-07
USP11	2.267834522	5.47469E-08
USP39	2.214987797	2.89526E-11
USP5	2.206641088	4.79505E-13
USP36	2.155383147	6.75756E-08
USP37	2.140295652	6.80989E-06
USP42	2.004362934	3.87037E-07
USP33	1.970531922	3.21092E-05
USP24	1.903860951	4.44187E-05
USP40	1.86603239	5.15633E-08
USP19	1.84826459	7.98074E-09
USP51	1.829917148	0.181272525
USP6	1.788081246	0.000309536
USP30	1.778839229	1.28833E-06
USP16	1.756498948	2.89561E-05
USP28	1.753369982	3.0596E-05
USP34	1.748864366	2.44737E-06
USP7	1.715657352	2.8628E-08
USP32	1.678044268	0.000160033
USP4	1.633647317	9.07146E-06
USP13	1.610067084	0.000530334
USP48	1.605525165	5.94223E-07
USP8	1.603138348	3.8408E-05
USP20	1.59637505	3.38606E-08
USP45	1.593446948	0.00163141
USP47	1.515399433	0.000118691
USP25	1.511177673	0.00197186
USP12	1.444762184	0.409306427
USP53	1.420120437	0.32520132

USP10	1.415685614	0.000932035
USP9X	1.413418676	0.000350617
USP2	1.388377328	0.856827092
USP9Y	1.364606219	0.367532674
USPL1	1.266937596	0.120210293
USP15	1.201895041	0.240381614
OTUD4	1.174323133	0.449080733
USP6NL	1.166292669	0.198634372
USP18	1.159572212	0.381991178
USP50	1	0.787503181
USP38	0.993233066	0.194152828
USP44	0.847593718	0.069908955
USP43	0.846289658	0.312404287
USP17	NA	NA
USP17L2	NA	NA
USP17L6P	NA	NA
USP26	NA	NA
USP29	NA	NA
**OTU family**		
OTUB2	7.188493266	1.49632E-09
OTUD7A	3.398297586	9.92464E-06
OTUD6B	3.308460973	2.21158E-08
OTUD3	2.828913095	8.07502E-05
OTUD7B	2.453160651	5.84104E-06
ALG13	2.112971111	0.000001
OTUB1	2.088885875	4.47514E-10
OTULIN	2.015745576	0.000001
VCPIP1	1.676041636	0.000167363
ZRANB1	1.312428885	0.153661456
OTUD1	1.058225329	0.439996236
OTUD4	1.174323133	0.449080733
OTUD5	1.553118509	3.47409E-07
OTUD6A	NA	NA
**JAMM family**		
PSMD14	2.054390897	8.66839E-07
PSMD7	2.153836181	1.70315E-06
PRPF8	2.088065448	0.000358897
MPND	1.843564189	2.01357E-05
MYSM1	1.577777778	0.000219769
COPS6/CSN6	1.308472021	3.27852E-14
STAMBPL1	0.453117352	2.76123E-06
STAMBP	1.573292978	1.12825E-06
BRCC3	1.234379562	1.15454E-06

COPS5	1.263253397	1.53699E-10
EIF3F	1.168754755	2.29492E-08
EIF3H	1.594306541	6.65907E-08
**MCPIP family**		
ZC3H12A	1.051391466	0.14600871
ZC3H12B	0.517522124	0.030769727
ZC3H12C	1.130651558	0.837094549
ZC3H12D	1.608	0.013614281
NYNRIN	1.414326466	0.150998298
KHNYN	0.964744058	3.96851E-08
N4BP1	1.532289442	0.000332133
**UCH family**		
UCHL1	75.09866667	0.066840399
UCHL3	3.15745614	0.11464283
UCHL5	1.661445244	7.52876E-07
BAP1	1.012614207	2.50874E-11
**MJD family**		
ATXN3	0.962463768	0.001394892
JOSD1	0.70458079	5.22817E-05
JOSD2	1.006901141	3.36E-08

Supplemental table 2. The fold change (FC) of relative protein level of OTU DUBs in human HCC tissues and the non-tumor liver tissues.

NO.	OTUB1	OTUD4	OTUD5	OTUD6B	OTUD7B	OTULIN	VCPIP1	ALG13
1	9.04	T[a].only	T.only	T.only	T.only	T.only	T.only	T.only
2	6.90	T.only	T.only	T.only	T.only	T.only	T.only	T.only
3	6.55	T.only	T.only	T.only	T.only	T.only	T.only	T.only
4	6.25	T.only	T.only	T.only	T.only	T.only	327.82	T.only
5	5.87	T.only	T.only	T.only	T.only	T.only	175.47	T.only
6	5.31	T.only	T.only	T.only	T.only	T.only	86.73	T.only
7	5.18	T.only	T.only	T.only	T.only	T.only	73.36	T.only
8	4.89	T.only	NA	T.only	T.only	T.only	44.53	T.only
9	4.88	T.only	NA	T.only	T.only	T.only	31.82	T.only
10	4.68	T.only	NA	T.only	T.only	T.only	27.11	T.only
11	4.35	T.only	NA	T.only	T.only	T.only	20.89	T.only
12	4.21	T.only	NA	T.only	T.only	T.only	19.15	T.only
13	4.19	T.only	NA	T.only	T.only	T.only	18.98	T.only
14	4.12	T.only	NA	T.only	T.only	T.only	18.91	T.only
15	3.82	T.only	NA	T.only	T.only	T.only	18.30	P.only
16	3.73	T.only	NA	T.only	T.only	T.only	18.14	P.only
17	3.69	T.only	NA	T.only	T.only	T.only	17.28	P.only
18	3.33	T.only	NA	T.only	T.only	P[b].only	15.75	P.only
19	3.23	T.only	NA	T.only	T.only	NA	15.37	P.only
20	3.22	T.only	NA	T.only	T.only	66.60	14.88	P.only
21	3.15	T.only	NA	T.only	T.only	29.66	14.61	P.only
22	3.14	T.only	NA	T.only	T.only	27.95	12.17	P.only
23	3.11	T.only	NA	T.only	T.only	23.85	11.68	P.only
24	2.92	T.only	NA	T.only	T.only	20.72	9.80	P.only
25	2.90	T.only	NA	T.only	T.only	19.37	9.43	P.only
26	2.90	T.only	NA	T.only	T.only	13.93	9.31	P.only
27	2.82	T.only	NA	T.only	T.only	11.38	9.25	P.only
28	2.75	T.only	NA	T.only	T.only	10.21	9.12	P.only
29	2.72	T.only	NA	T.only	T.only	10.17	8.94	P.only
30	2.70	T.only	NA	T.only	T.only	7.74	8.92	P.only
31	2.68	T.only	NA	T.only	T.only	7.54	8.85	P.only
32	2.68	T.only	NA	T.only	T.only	7.30	8.21	NA
33	2.64	T.only	NA	T.only	T.only	7.29	8.07	NA
34	2.58	T.only	NA	T.only	T.only	7.17	7.86	NA
35	2.45	T.only	NA	T.only	T.only	6.31	7.56	NA
36	2.32	T.only	NA	T.only	T.only	5.93	7.53	NA
37	2.31	T.only	NA	T.only	T.only	5.62	7.14	50.16
38	2.31	T.only	NA	T.only	T.only	5.26	7.10	39.50

39	2.30	T.only	NA	T.only	P.only	5.14	6.91	23.87
40	2.26	P.only	NA	T.only	P.only	4.90	6.87	16.60
41	2.25	P.only	NA	T.only	P.only	4.62	6.76	16.36
42	2.24	P.only	NA	T.only	NA	4.55	6.71	14.77
43	2.16	P.only	NA	T.only	NA	4.31	6.51	10.37
44	2.10	P.only	NA	T.only	74.92	4.28	6.28	9.19
45	2.09	P.only	NA	T.only	51.76	4.08	6.23	8.99
46	2.07	P.only	NA	T.only	42.10	3.84	6.23	8.96
47	2.06	P.only	NA	T.only	26.85	3.75	5.96	8.62
48	2.04	P.only	NA	T.only	26.52	3.37	5.76	8.61
49	2.02	P.only	NA	T.only	18.11	3.11	5.49	7.52
50	1.77	P.only	NA	T.only	17.41	2.95	5.28	6.79
51	1.76	P.only	NA	T.only	16.35	2.73	5.23	5.83
52	1.74	P.only	NA	T.only	15.20	2.55	5.18	5.73
53	1.73	NA	NA	T.only	12.82	2.55	4.93	5.68
54	1.72	NA	NA	T.only	12.61	2.55	4.90	5.41
55	1.71	NA	NA	T.only	11.28	2.48	4.61	5.32
56	1.68	NA	NA	T.only	11.22	2.40	4.43	4.98
57	1.64	NA	NA	T.only	10.46	2.39	4.13	4.79
58	1.62	NA	NA	T.only	10.16	2.33	4.12	4.30
59	1.61	NA	NA	T.only	9.88	2.20	4.11	3.98
60	1.61	NA	NA	T.only	9.41	2.16	4.06	3.71
61	1.60	NA	NA	T.only	8.85	2.14	3.73	3.65
62	1.58	NA	NA	T.only	7.90	2.10	3.61	3.40
63	1.54	NA	NA	P.only	7.43	2.07	3.47	3.31
64	1.54	NA	NA	P.only	7.07	2.05	3.47	2.49
65	1.54	NA	NA	P.only	6.77	2.01	3.38	2.43
66	1.53	NA	NA	P.only	6.68	1.89	3.34	2.36
67	1.53	NA	NA	P.only	6.51	1.87	3.27	2.30
68	1.52	NA	NA	NA	6.45	1.81	2.83	2.16
69	1.52	NA	NA	NA	5.98	1.79	2.75	1.91
70	1.51	NA	NA	NA	5.98	1.76	2.70	1.89
71	1.50	63.58	NA	NA	5.83	1.73	2.61	1.86
72	1.48	32.05	NA	NA	5.67	1.61	2.60	1.85
73	1.47	14.05	NA	NA	5.59	1.59	2.58	1.84
74	1.47	11.61	NA	NA	5.53	1.59	2.57	1.60
75	1.45	6.57	NA	NA	5.26	1.58	2.57	1.59
76	1.43	6.45	NA	NA	5.07	1.57	2.50	1.55
77	1.37	5.33	NA	NA	4.95	1.50	2.50	1.49
78	1.36	3.89	NA	NA	4.82	1.48	2.41	1.40
79	1.35	3.75	NA	NA	4.53	1.47	2.24	1.39
80	1.33	3.21	NA	NA	4.24	1.44	2.20	1.33
81	1.32	3.18	NA	NA	4.13	1.39	2.12	1.20

82	1.32	2.87	NA	NA	3.96	1.38	1.98	1.14
83	1.31	2.82	NA	NA	3.81	1.29	1.90	1.13
84	1.31	2.76	NA	NA	3.52	1.28	1.86	1.06
85	1.27	2.32	NA	NA	3.46	1.27	1.85	1.05
86	1.24	2.25	NA	NA	3.41	1.24	1.79	0.98
87	1.22	2.05	NA	NA	3.15	1.20	1.74	0.91
88	1.22	1.95	NA	NA	3.11	1.18	1.73	0.90
89	1.18	1.80	NA	NA	3.05	1.18	1.67	0.75
90	1.17	1.54	NA	NA	3.02	1.17	1.63	0.75
91	1.17	1.50	NA	NA	2.61	1.16	1.62	0.71
92	1.16	1.38	NA	NA	2.40	1.12	1.61	0.66
93	1.16	1.26	NA	NA	2.19	1.10	1.59	0.66
94	1.14	1.26	NA	NA	2.12	1.05	1.50	0.62
95	1.07	1.21	NA	24.97	2.04	1.05	1.32	0.60
96	1.05	1.15	NA	16.78	2.03	0.98	1.31	0.58
97	1.05	1.13	NA	8.58	1.95	0.98	1.23	0.57
98	1.05	1.11	NA	8.16	1.89	0.95	1.21	0.52
99	1.04	0.97	NA	7.16	1.81	0.95	1.21	0.51
100	1.04	0.83	NA	7.13	1.72	0.94	1.18	0.46
101	0.98	0.83	NA	5.41	1.60	0.93	1.10	0.45
102	0.98	0.78	NA	5.16	1.53	0.92	1.00	0.42
103	0.97	0.74	NA	4.18	1.34	0.79	0.89	0.41
104	0.97	0.71	NA	3.84	0.91	0.78	0.86	0.39
105	0.95	0.71	NA	3.81	0.83	0.71	0.80	0.38
106	0.92	0.63	NA	3.22	0.82	0.63	0.80	0.38
107	0.88	0.52	NA	2.99	0.60	0.52	0.56	0.37
108	0.84	0.36	NA	2.50	0.60	0.50	0.52	0.35
109	0.70	0.20	NA	2.14	0.51	0.42	0.47	0.32
110	0.65	0.12	NA	1.32	0.51	0.38	0.24	0.27
111	0.42	0.05	NA	0.65	0.33	0.36	0.04	0.09

[a] Tumor; [b] Peri-tumor

Supplemental table 3. Clinicopathological significance of OTUD6B in human HCC tissues.

Clinicopathological indexes		No. of patients (%) （n=89）	Tumor OTUD6B Protein level			
			Low(%)（n=18)	mid(%)（n=52)	High(%) (n=19)	*p* value
Age(year)	≤50	40(44.9)	11(61.1)	20(38.5)	9(47.4)	0.4181
	>50	49(55.1)	7(38.9)	32(61.5)	10(52.6)	
Gender	Female	10(11.2）	2(11.1）	5(9.62)	3(15.8)	0.644
	Male	79(88.8）	16(88.9)	47(90.4)	16(84.2)	
HBsAg	Negative	18(20.5)	3(16.7)	10(19.2)	5(26.3)	0.4642
	Positive	70(79.5)	15(83.3)	41(78.8)	14(73.7)	
HBcAb	Negative	7(8.1)	2(11.1)	4(7.7)	1(5.3)	0.5422
	Positive	79(91.9)	16(88.9)	46(88.5)	17(89.5)	
AFP (ng/ml)	≤20	35(39.8)	2(11.1)	27(51.9)	6(31.6)	0.2262
	>20	53(60.2)	16(88.9)	24(46.2)	13(68.4)	
GGT (U/L)	≤54	51(58.0)	8(44.4)	27(51.9)	16(84.2)	0.0134
	>54	37(42.0)	10(55.6)	24(46.2)	3(15.8)	
Liver cirrhosis	No	9(10.2)	2(11.1)	5(9.6)	2(10.5)	0.4408
	yes	79(89.8)	16(88.9)	31(59.6)	32(168.4)	
Tumor size(cm)	≤5	62(69.7)	12(66.7)	34(65.4)	16(84.2)	0.2374
	>5	27(30.3）	6(33.3)	18(34.6)	3(15.8)	
Tumor number	Single	78(87.6)	15(83.3)	46(88.5)	17(89.5)	0.5746
	Multiple	11(12.4)	3(16.7)	6(11.5)	2(10.5)	
Microvascular invasion	absence	57(73.1)	11(61.1)	32(61.5)	14(73.7)	0.5395
	present	21(26.9)	4(22.2)	14(26.9)	3(15.8)	
Tumor encapsulation	complete	41(46.6)	7(38.9)	25(48.1)	9(47.4)	0.6131
	none	47(53.4)	11(61.1)	26(50)	10(52.6)	
Histological grade	I+II	42(47.7)	5(27.8)	26(50)	11(57.9)	0.0921
	III+IV	46(52.3)	12(66.7)	26(50)	8(42.1)	
TNM stage	I	57(64.0)	11(61.1)	32(61.5)	13(68.4)	0.4884
	II+III	32(36.0)	7(38.9)	20(38.5)	5(26.3)	
Recurrence	No	40(44.9)	6(33.3)	21(40.4)	13(68.4)	0.0304
	Yes	49(55.1)	12(66.7)	31(59.6)	6(31.6)	
Death	No	57(64.0)	9(50)	31(59.6)	17(89.5)	0.0117
	Yes	32(36.0)	9(50)	21(40.4)	5(26.3)	

Supplemental table 4. Differential gene expression in HCC cells with OTUD6B knockdown and NTC

Gene ID	Ctrl FPKM	shOTUD6B FPKM	log2(shOTUD6B/Ctrl)	Qvalue(Ctrl-vs-shOTUD6B)	Pvalue(Ctrl-vs-shOTUD6B)
100529144	0.42	0.97	1.20	1.25E-12	3.66E-14
100532726	0.01	0.20	4.55	1.03E-07	5.64E-09
10098	0.01	0.11	3.81	4.47E-06	3.29E-07
101060179	0.26	0.66	1.36	3.78E-05	3.47E-06
10178	0.01	0.04	3.30	0.000523143	6.88E-05
10215	0.01	0.41	6.19	3.97E-17	7.37E-19
102723722	0.40	1.16	2.64	3.35E-23	3.80E-25
102724994	0.12	0.45	2.01	8.81E-09	4.16E-10
10278	0.00	0.08	5.00	0.000309659	3.75E-05
10391	0.01	0.26	4.37	9.89E-14	2.57E-15
10395	0.01	0.09	3.21	6.39E-07	4.06E-08
10485	0.09	0.53	2.52	6.02E-06	4.57E-07
10631	0.07	1.55	4.78	5.68E-81	1.44E-83
116448	0.02	0.40	4.44	1.82E-14	4.33E-16
1462	0.00	0.13	6.83	3.64E-13	1.00E-14
1634	0.70	1.97	1.49	1.28E-20	1.84E-22
165	0.04	0.16	1.94	4.35E-05	4.07E-06
1809	0.01	0.09	2.93	6.69E-06	5.15E-07
1848	0.47	1.22	1.38	4.38E-18	7.64E-20
220382	0.00	0.12	5.00	0.000309659	3.75E-05
221981	0.00	0.03	5.09	0.000183799	2.06E-05
2290	0.01	0.11	3.59	4.01E-05	3.73E-06
23266	0.00	0.05	4.25	0.000128334	1.38E-05
23302	0.01	0.09	3.09	3.28E-06	2.35E-07
2335	0.07	0.23	1.69	1.05E-10	3.74E-12
23767	0.01	0.09	3.17	3.43E-05	3.13E-06
2583	0.01	0.08	4.57	1.00E-05	8.02E-07
2841	0.05	0.18	2.05	0.000676782	9.36E-05
28951	0.01	0.31	5.02	2.52E-22	3.19E-24
2953	0.12	0.71	2.63	4.28E-09	1.93E-10
3400	0.00	0.12	5.09	3.33E-08	1.72E-09
341640	0.08	0.25	1.65	6.70E-23	7.71E-25
3485	0.09	2.09	4.89	4.07E-47	2.02E-49
3488	0.02	0.16	3.15	1.59E-12	4.74E-14
357	0.01	0.11	3.13	1.93E-06	1.31E-07
3690	0.04	0.15	2.03	4.73E-06	3.51E-07
3737	0.00	0.03	5.50	1.00E-05	7.97E-07
3800	0.00	0.06	4.80	3.11E-07	1.86E-08

4133	0.02	0.14	2.49	5.78E-08	3.06E-09
4239	0.06	0.29	2.22	4.48E-05	4.22E-06
4313	0.03	0.13	2.32	0.000258269	3.04E-05
4323	0.06	0.41	2.91	5.82E-17	1.10E-18
441519	0.13	0.64	2.34	4.53E-06	3.35E-07
4684	0.03	0.11	1.93	0.000105985	1.11E-05
4811	0.01	0.12	4.03	1.63E-10	6.03E-12
4843	0.02	0.33	3.72	2.55E-18	4.40E-20
5098	0.01	0.22	4.23	2.54E-14	6.19E-16
51299	0.00	0.12	4.81	0.000896824	0.000128772
5156	0.03	0.13	2.44	1.51E-07	8.59E-09
51704	0.01	0.14	3.86	2.58E-06	1.79E-07
53826	0.07	0.52	3.04	3.21E-11	1.10E-12
5396	0.00	0.05	4.81	0.000881027	0.000126427
5453	0.03	0.15	2.34	0.000226626	2.62E-05
56140	0.05	0.14	1.44	0.000571728	7.66E-05
5625	0.23	0.72	1.75	1.21E-09	5.14E-11
56265	0.01	0.16	4.70	2.77E-06	1.96E-07
5629	0.00	0.02	4.81	0.000881027	0.000126427
56999	0.03	0.12	2.02	3.00E-07	1.79E-08
5803	0.02	0.63	4.86	2.84E-59	1.09E-61
5806	0.00	0.11	4.91	0.000522998	6.87E-05
6299	0.00	0.04	4.81	0.000881027	0.000126427
6507	0.01	0.23	4.42	3.19E-14	7.83E-16
653781	0.03	0.13	2.29	0.000906046	0.000130175
6657	0.06	0.54	3.25	5.81E-17	1.09E-18
6664	0.00	0.04	4.46	2.51E-05	2.19E-06
6678	0.10	1.50	3.87	1.79E-77	5.00E-80
6696	0.05	0.29	2.77	1.20E-05	9.78E-07
6925	0.05	0.22	2.08	3.70E-11	1.27E-12
7070	0.01	0.38	4.81	6.21E-13	1.77E-14
7345	0.06	0.71	3.49	7.75E-11	2.75E-12
752014	0.00	0.21	5.34	3.40E-05	3.07E-06
79870	0.03	0.18	2.89	2.56E-06	1.78E-07
81557	0.03	0.15	2.70	0.000207786	2.37E-05
81848	0.14	0.36	1.33	4.06E-08	2.14E-09
8293	0.61	6.68	2.92	8.52E-101	1.49E-103
8362	0.84	2.60	1.64	3.81E-05	3.51E-06
8404	0.05	0.39	3.12	1.44E-13	3.77E-15
8406	0.03	0.18	2.88	0.000460084	5.90E-05
84446	0.06	0.21	1.86	0.000246847	2.88E-05
85027	0.02	0.16	2.76	0.000125215	1.34E-05
8642	0.01	0.04	3.00	9.51E-06	7.53E-07

8671	0.05	0.19	2.03	6.12E-10	2.53E-11
8728	0.00	0.10	5.46	1.49E-10	5.47E-12
8840	0.00	0.05	5.00	0.000309659	3.75E-05
894	0.01	0.16	4.17	1.74E-15	3.72E-17
9500	0.22	0.56	1.36	7.15E-08	3.86E-09
9537	0.61	0.99	1.11	2.33E-08	1.18E-09
9715	0.09	0.29	1.74	2.07E-08	1.03E-09
9865	0.00	0.09	4.96	1.75E-07	1.00E-08
9900	0.03	0.18	2.88	2.31E-07	1.34E-08

Supplemental table 5. List of primers used for plasmids construction and target sequence of shRNAs.

Genes	Forward Primer 5'-3'	Reverse Primer 5'-3'
**Eukaryotic expression vector (pcDNA3.1-CMV backbone)**		
Flag-OTUD6B	CCCAAGCTTATGGAGCCCC GGGTGAG	CGCGGATCCTTAGCTGCAATT TTCAGTAACT
Flag-OTUD6B-△OTU	CCCAAGCTTATGGAGCCCC GGGTGAG	CGCGGATCCTTACACCAAGTT TGAAATGT
Flag-OTUD6-△N	CCCAAGCTT CTTGAGAATCAGCCACC	CGCGGATCCTTAGCTGCAATT TTCAGTAACT
Flag-OTUD6-C188S	CTATGTATAAAGCCATTGA AGATCAACT	AGTGGCCATCAGATGGAATCT GT
Flag-OTUD6-C17S	AGCAGGTTTCTTCTAGCGC GTGTGC	CCGGCTAGTAGGCACCTTCCA C
Flag-OTUD6-ASA	GCTTATAATTCGGTTACAC GGTTG	TTCTCCTAAGCCATATGCATG TC
Myc-pVHL-N	CGGAATTCGGATGCCCCGG AGGGCGG	GGGGTACCTCATGGCAGTGTG ATATTGGCAAAAAT
Myc-pVHL-C	CGGAATTCGGGTGTATACT CTGAAAGAGC	GGGGTACCTCAATCTCCCATC CGTTGATGTG
Myc-WSB1	GGAATTCGGATGGCCAGCT TTCCCCCGAGGGT	CGGGGTACCCTAAATACGATA CGAGAGAAACTCC
Myc-elonginB	CGGAATTCGGATGGACGTG TTCCTCATGATCC	GGGGTACCTCACTGCACGGCT TGTTCATTG
Myc-elonginC	ACGCGTCGACCATGGATGG AGAGGAGAAAACCT	GGGGTACCTTAACAATCTAAG AAGTTCGCAGCC
**Prokaryotic expression vector (pET-28a-His or pGEX-6P-1-GST)**		
His-OTUD6B	CGCGGATCCATGGAGCC	CCCAAGCTTTTAGCTGCAAT
His-WSB1	CCGGAATTCATGGCCAGCT TTCCCCCGAG	ACGCGTCGACCTAAATACGAT ACGAGAGAAACT
GST-OTUD6B	CGCGGATCCATGGAGCCCC GGGT	ACGCGTCGACTTAGCTGCAAT TTTCAGTAACT
GST-pVHL	CGCGGATCCATGCCCGGAG GGC	ACGCGTCGACTCAATCTCCCA TCCGTTGAT
GST-elonginB	CGCGGATCCATGGACGTGT TCCTCATGATCC	ACGCGTCGACTCACTGCACGGC TTGTTCATTG
**Target sequences of shRNA**		
	#1	#2
shOTUB1	CACCACCAATCCGCACATC TT	TGTGGTTGTAAATGGTCCTAT
shOTUB2	CCGTTTACCTGCTCTATAA	CTTCTGCACTCACGAAGTA

shOTUD2	TGGTATGTCAGAAAGGATT	AGCAGATGAAGCTAGAAGA
shOTUD3	TGGAAATCAGGGCTTAAAT	GAGTTACACATCGCATATC
shOTUD4	ACGAAAAGACAAAGACTCT	AGGTTATCAGTACCATCGA
shOTUD5	AGATGCTAGAAGACAAGAA	ACCTAGACAGTATGAAGAA
shOTUD6B	GGTATTGACCGAAGAGCTTGA	GCTGAGAAGGCATCGCAAAGA
shOTUD7B	GCAGCAGACACAGCAGAAT	TGGTATACACAGAAGATGA
shOTULIN	ATCACCACGGACTCGCCGTA	ACCACGGACTCGCCGTATGG
shHIF-1	GTTACGTTCCTTCGATCAG	/
shHIF-2	CGACCTGAAGATTGAAGTGAT	/
shpVHL	GGAAAATACACGTGGGGTGG	/
shNTC	TTCTCCGAACGTGTCACGT	/

Supplemental table 6. List of primary antibodies.

Name	Cat#	Company	Dilution rate
OTUB1	ab175200	Abcame	1:1000 for WB
OTUB2	GTX83953	GeneTex	1:1000 for WB
OTUD2	HPA028400	Atlas	1:600 for WB
OTUD3	HPA028544	Atlas	1:500 for WB
OTUD4	MA5-31812	ThermoreFisher	1:800 for WB
OTUD5	HPA017375	Atlas	1:600 for WB
OTUD6B	NBP1-85652	Novas	1:800 for WB and IF; 1:150 for IHC
OTUD7B	16605-1-AP	Proteintech	1:2000 for WB
OTULIN	14127s	CST	1:1000 for WB
E-Cadherin	Ab1416	Abcame	1:1000 for WB
Snail	ab53519	Abcame	1:1000 for WB
HIF-1α	ab1	Abcam	1:800 for WB; 1:50 for Chip
HIF-2α	ab199	Abcam	1:800 for WB; 1:50 for Chip
HIF-1β		Abcam	1:50 for Chip
pVHL	NB100-485	Novas	1:500 for WB; 1:150 for IHC
pVHL	sc-55506	Santa Cruz	1:500 for IF
elonginB	ab168836	Abcam	1:1000 for WB
elonginB	sc-133090	Santa Cruz	1:500 for IF
elonginC	ab226831	Abcam	1:1000 for WB
Actin	sc-1616	Santa Cruz	1:1000 for WB
GAPDH	sc-25778	Santa Cruz	1:1000 for WB
Tubulin	TA-10	Zsbio	1:2500 for WB
HA	M180-3	MBL	1:2500 for WB
Myc	sc-374171	Santa Cruz	1:2500 for WB
Flag	sc-965	Santa Cruz	1:2500 for WB
His	sc8036	Santa Cruz	1:2500 for WB

Supplememntal table 7. List of real-time Q-PCR primers.

Gene name	Forward Primer 5'-3'	Reverse Primer 5'-3'
OTUD6B	TGAGAAGGCATCGCAAAGAGA	ATCTTCGGTGAGTTGCTTCCT
OTUD6A	ATGGATGATCCGAAGAGTGAACA	GGTCTTGGGGACCGAGTTTT
HIF-1α	TTCCCGACTAGGCCCATTC	CAGGTATTCAAGGTCCCATTTCA
HIF-2α	CGGAGGTGTTCTATGAGCTGG	AGCTTGTGTGTTCGCAGGAA
VEGF	GAGGAGCAGTTACGGTCTGTG	TCCTTTCCTTAGCTGACACTTGT
LOXL2	GGGTGGAGGTGTACTATGATGG	CTTGCCGTAGGAGGAGCTG
MMP2	TGACTTTCTTGGATCGGGTCG	AAGCACCACATCAGATGACTG
MMP14	GGCTACAGCAATATGGCTACC	GATGGCCGCTGAGAGTGAC
DCN	GACAAGGTCCGCCAGTTTATG	TCGTCTAGTCTCCACTCATTCTG
VCAN	GTAACCCATGCGCTACATAAAGT	GGCAAAGTAGGCATCGTTGAAA
NOS2	TGTGGAAGGACTACTTCAACCT	GGGACTTGGCTCCTCAATCT
pVHL	GGGAACGGGGTGGGTTTAG	GCTCGCGTGAGTTCACAGA
VEGF-HRE	ATCCGTCCTCAAGTTGCTTGT	TCGATCTCAGGCTATCTCGTG
OTUD6B-HRE	AATCCAGCGTGCGCCCTTCTGTTAA	CTCAATCCTTCTTATATCTTGTTTCC
Actin	CATCCTCACCCTGAAGTACCC	AGCCTGGATAGCAACGTACATG
GAPDH	TGCACCACCAACTGCTTAGC	GGCATGGACTGTGGTCATGAG
